# Azaphilones produced by *Penicillium maximae* with their cell death-inducing activity on Adriamycin-treated cancer cell

**DOI:** 10.1186/s41021-023-00261-w

**Published:** 2023-01-19

**Authors:** Takahiro Matsumoto, Erika Ohnishi, Takahiro Kitagawa, Masaya Okayama, Youhei Saito, Hayato Yoshikawa, Tomoe Ohta, Tatsusada Yoshida, Yuji Nakayama, Tetsushi Watanabe

**Affiliations:** 1 Kyoto Pharmaceutical University, 1 Misasagi-Shichono-cho, Yamashina-ku, 607-8412 Kyoto, Japan; 2 Faculty of Pharmaceutical Sciences, Nagasaki International University, 2825-7 Huis Ten Bosch-Cho, 859-3298 Sasebo, Nagasaki Japan

**Keywords:** *Penicillium maximae*, Azaphilone, Maximazaphilone, Time-lapse imaging, Heat shock protein, JKYM-AK1, Adriamycin, Fungus

## Abstract

**Background:**

Heat shock proteins (Hsps) are overexpressed in several tumors and contribute to cell proliferation, metastasis, and anticancer drug resistance. Therefore, Hsp inhibitors have enhanced cytotoxicity as chemotherapeutic agents and may be effective with a reduced dosage for tumor therapy to avoid side effects.

**Results:**

Four new azaphilones, maximazaphilones I–IV (**1**–**4**), and three known compounds (**5**–**7**) have been isolated from the airborne-derived fungus *Penicillium maximae*. Inhibitory effects of isolated compounds against induction of Hsp105 were evaluated by the luciferase assay system using Hsp105 promoter. In this assay, **2**–**4**, **6**, and **7** significantly inhibited *hsp105* promoter activity without cytotoxicity. In addition, all isolated compounds except for **5** significantly induced the death of Adriamycin (ADR)-treated HeLa cells. Interestingly, **1**–**4**, **6**, and **7** didn’t show anti-proliferative and cell death-inducing activity without ADR.

**Conclusion:**

This study revealed the chemical structures of maximazaphilones I–IV (**1**–**4**) and the potency of azaphilones may be useful for cancer treatment and reducing the dose of anticancer agents. In addition, one of the mechanisms of cell death-inducing activity for **2**–**4**, **6**, and **7** was suggested to be inhibitory effects of Hsp105 expression.

**Supplementary Information:**

The online version contains supplementary material available at 10.1186/s41021-023-00261-w.

## Introduction

Heat shock proteins (Hsps) play an important role in cellular homeostasis in response to stressors such as hypoxia, anoxia, high temperature, and several chemical agents that induce protein denaturation [1]. Hsps are overexpressed in several tumors and contribute to cell proliferation, metastasis, and anticancer drug resistance [2]. They are classified as Hsp27, Hsp40, Hsp60, Hsp70, Hsp90, and Hsp105 based on their molecular weights [3]. The anti-apoptotic function of Hsp70 and Hsp90 had been suggested to be one of the major mechanisms of chemotherapeutic agent resistance on cancer cells [4, 5]. Therefore, several Hsp70 and Hsp90 inhibitors are anticancer agents. Gambogic acid from *Garcinia harburyi* [6], derrubone from *Derris robusta* [7], and several azaphilones from *Aspergillus deflectus* [8] have been reported as Hsp90 inhibitors extracted from natural sources. A recent study revealed that decreased expression of Hsp105 enhances apoptosis induction against DNA-damaging agents, such as Adriamycin (ADR) [9]. However, the number of reported inhibitors of Hsp105 is limited [10, 11]. Considering these factors, our research group explored new inhibitors of Hsp expression, especially Hsp105.

To explore new Hsp expression inhibitors we selected azaphilones, secondary metabolites of fungi. Azaphilones have been isolated from *Penicillium* [12], *Aspergillus* [8], *Chaetomium* [13], *Talaromyces* [14], and *Muyocopron* [15] species. Among these fungi, more than 50 azaphilones were reported from the species of *Penicillium* section *Sclerotiora* [16–19] proving this species is the major source of azaphilones. Thus, we selected *Penicillium maximae* [20, 21] belonging to *Penicillium* section *Sclerotiora* from our library of airborne-derived fungi in Kyoto. In this study, we report chemical structures and inhibitory effects on Hsp expression of azaphilones produced by *P. maximae* JKYM-AK1.

## Materials and methods

### General experimental procedures

Specific rotations were obtained by using a JASCO P-2200 digital polarimeter (l = 5 cm). ECD spectroscopy was recorded using a JASCO J-1500 spectrometer. FAB-MS and HR-FAB-MS were recorded by using a JEOL JMS-SX 102 A mass spectrometer. ^1^ H NMR spectroscopy was recorded on JEOL ECS400 (400 MHz) and JNM-ECA 600 (600 MHz) spectrometers. ^13^ C NMR spectroscopy was recorded on a JNM-ECA 600 (150 MHz) spectrometer. 2D-NMR experiments were carried out on a JEOL JNM-ECA 600 (600 MHz) spectrometer.

Normal phase silica gel column chromatography was carried out using Silica gel 60 (Kanto Chemical Co., Inc. 63–210 mesh), and reversed phase silica gel column chromatography was carried out using C_18_-OPN (Nacalai Tesque Co., Inc. 140 μm). High-performance liquid chromatography (HPLC) was performed using a Shimadzu SPD-M20A UV–vis detector, Shimadzu LC-20AD pump, and Shimadzu SIL-20 A auto-injector. COSMOSIL 5C18-MS-II (Nacalai Tesque Co., Inc. 250 × 4.6 mm i.d., 250 × 10 mm i.d., and 250 × 20 mm i.d.) columns were used for analytical and preparative work.

### Collection and identification of the JKYM-AK1 strain

Airborne particles (PM_10_, aerodynamic diameter ≤ 10 μm) were collected in the city of Kyoto (135.81°E, 34.99°N) using a high-volume air sampler (HV1000R, Shibata Scientific Technology, Soka, Japan) equipped with an impactor (Shibata Scientific Technology) in September 2021. The collected airborne particles were immediately suspended in distilled H_2_O. The suspension was inoculated with potato dextrose agar (PDA) and chloramphenicol (0.1 g/L) for incubating at 28 °C for 3 days. The JKYM-AK1 strain was isolated as colonies and stored in 10% glycerin at − 80 °C. Using internal transcribed spacer 1 (ITS1, 5′-TCCGTAGGTGAACCTGCGG-3′) and ITS4 (5′-TCCTCCGCTTATTGATATGC-3′) as primers, the ITS1-5.8 S-ITS2 sequence region (511 base pairs, GenBank accession number ON150838) of JKYM-AK1 was obtained. BLAST analysis (NCBI database) showed that the sequence has a 99.60% identity of *Penicillium maximae* (Accession number: EU427298).

### Fermentation and extraction

The seed culture of the fungus was prepared in a potato dextrose medium and incubated at 28 °C for 5 days at 200 rpm. The seed culture was added to 2 L × 20 Erlenmeyer flasks containing potato dextrose medium (1 L). The cultures were further incubated at 28 °C for 20 days followed by filtration. Mycelia was extracted three times with methanol by refluxing for 1 h. The solvent was evaporated to obtain a methanol extract. The methanol extract was combined with the supernatant and partitioned using EtOAc–water mixture.

### Isolation of the compounds produced by the JKYM-AK1 strain

The EtOAc soluble fraction (8.31 g) was purified by normal phase silica gel column chromatography [*n*-hexane–CHCl_3_ (1:1 → 1:4→ 0:1, v/v) → CHCl_3_–MeOH (50:1 → 30:1 → 20:1 → 10:1 → 5:1 → 1:1, v/v)] and ten fractions were collected. Fraction 4 (2.3 g) was further separated using reversed phase silica gel column chromatography and eight fractions were collected. Fraction 4 − 3 (73.3 mg) was purified using HPLC {H_2_O–CH_3_CN (30:70, v/v)} to obtain **1** (3.2 mg), **2** (5.1 mg), **3** (8.4 mg), and **4** (7.1 mg). Fraction 4–4 (118.5 mg) was purified using HPLC {H_2_O–CH_3_CN (40:60, v/v)} to obtain **5** (48.9 mg). Fraction 5 (1.1 g) was separated using reversed phase silica gel column chromatography and ten fractions were obtained. Fraction 5 − 4 (22.2 mg) was purified using HPLC {H_2_O–CH_3_CN (60:40, v/v)} to obtain **7** (2.3 mg). Fraction 5–5 (23.3 mg) was purified using HPLC {H_2_O–CH_3_CN (65:35, v/v)} to obtain **6** (2.7 mg).

### Maximazaphilone I (1)

Yellow amorphous solid; [*α*]^25^_D_ +44.2 (*c* 0.16, MeOH); ^1^ H NMR (CDCl_3_, 600 MHz) and ^13^ C NMR (CDCl_3_, 150 MHz), see Table 1; positive-ion FAB-MS *m/z* 385 [M + H]^+^; HR-FAB-MS *m/z* 385.2018 [M + H]^+^ (calcd for C_23_H_29_O_5_, 385.2015).

### Maximazaphilone II (2)

Yellow amorphous solid; [*α*]^25^_D_ +100.3 (*c* 0.27, MeOH); ^1^ H NMR (CDCl_3_, 600 MHz) and ^13^ C NMR (CDCl_3_, 150 MHz), see Table 1; positive-ion FAB-MS *m/z* 319 [M + H]^+^; HR-FAB-MS *m/z* 319.1928 [M + H]^+^ (calcd for C_19_H_27_O_4_, 319.1909).

### Maximazaphilone III (3)

Yellow amorphous solid; [*α*]^25^_D_ +54.4 (*c* 0.21, MeOH); ^1^ H NMR (CDCl_3_, 600 MHz) and ^13^ C NMR (CDCl_3_, 150 MHz), see Table 1; positive-ion FAB-MS *m/z* 361 [M + H]^+^; HR-FAB-MS *m/z* 361.2025 [M + H]^+^ (calcd for C_21_H_29_O_5_, 361.2015).

### Maximazaphilone IV (4)

Yellow amorphous solid; [*α*]^25^_D_ +173.0 (*c* 0.15, MeOH); ^1^ H NMR (CDCl_3_, 600 MHz) and ^13^ C NMR (CDCl_3_, 150 MHz), see Table 1; positive-ion FAB-MS *m/z* 319 [M + H]^+^; HR-FAB-MS *m/z* 319.1919 [M + H]^+^ (calcd for C_19_H_27_O_4_, 319.1909).

### Calculation of theoretical ECD spectra for maximazaphilone I, II, and IV (1, 2, and 4)

A preliminary molecular mechanics (MM) conformational search was carried out on **1**, **2**, and **4** in vacuum by using the Merck molecular force field (MMFF) as implemented in Spartan’16 program [22]. The stable energy conformers of each compound with Boltzmann distributions over 1% were further optimized at the B3LYP/def2-TZVP level of density functional theory (DFT). The normal mode analysis was done at the same level to confirm none of the conformers showed imaginary frequencies and to obtain the Gibbs free-energies [23]. The low free-energy conformers for each compound with Boltzmann distributions over 1% (Fig. S 4) were subjected to the ECD calculations using time-dependent density functional theory (TD-DFT) at the B3LYP/def2-TZVPP level. Both for the DFT and TD-DFT calculations were performed using an integral equation formalism polarizable continuum model (IEFPCM) in MeOH using Gaussian 16 [24]. The resultant rotatory strengths of the lowest 30 excited states for each conformer were converted into Gaussian-type curves with half-bands (0.30 eV) using SpecDis v1.71 [25]. The calculated ECD spectra of each of **1**, **2**, and **4** were composed after correction based on the Boltzmann distribution of conformers and their relative free-energy.

### Cells

Human cervical carcinoma (HeLa) cells and mouse pGL105/C3H cells [26] were maintained in Dulbecco’s Modified Eagle Medium (DMEM) with low glucose (Wako Pure Chemical Industries, Osaka, Japan) supplemented with 5% fetal bovine serum (Merck, Darmstadt, Germany) under a 5% CO_2_ atmosphere at 37 ℃.

### Measurement of Luc activity

Stable *hsp105* promoter-luciferase reporter cell lines (pGL105/C3H cells) were cultured in a flat-bottomed 96-well plate (Coster 3596; Corning, NY, USA) and incubated to reach 70 − 80% confluence. The cells were pretreated with test compounds for 30 min and exposed to heat shock at 41℃ for 3 h using a water bath. After washing the cells with PBS (-) (Wako Pure Chemical Industries) twice, 150 µL of 1X Glo Lysis Buffer (Promega, Madison, WI, USA) was added to each well and mixed by shaking for 20 min at room temperature. Luciferase activity and cell viability were measured on the 96-well white plate (136,101; Thermo Fisher Scientific, Waltham, MA, USA) by using a luminometer (GloMax® Discover System; Promega). Luciferase assay reagent (Promega) and CellTiter-Glo® 3D reagent (Promega) were used for the measurement of luminescence and cell viability, respectively.

### Time-lapse imaging

Time-lapse imaging was performed using an Operetta high-content imaging system (PerkinElmer, Waltham, MA) as described previously [27]. The cells were cultured in a flat-bottomed 24-well plate (Coaster 3526; Corning) to reach 70 − 80% confluence. The cells were treated with test compounds or Adriamycin prior to the time-lapse cell imaging. Images were captured at 10 min intervals for 24 h under a 5% CO_2_ atmosphere at 37 ℃.

### Evaluation of Hsps expression by western blot analysis

HeLa cells (1.0 × 10^5^) were seeded in 35-mm dishes and cultured. After 24 h of incubation, the cells were treated with KRIBB11 or **6** for 30 min. Then, cells were exposed to 42 °C for 1 h for Hsps induction and were recovered at 37 °C for 5 h. Western blot analysis was performed as described previously [10]. Briefly, cells were lysed with SDS sample buffer and boiled at 100 °C for 5 min. Proteins were separated by SDS-PAGE and transferred to polyvinylidene difluoride membranes (Pall Corporation, Port Washington, NY, USA). Blots were incubated with Blocking One reagent (Nacalai Tesque, Kyoto, Japan) and sequentially incubated with appropriate primary and secondary antibodies. Chemiluminescence was detected with an LAS-4000 mini-image analysis system (Fujifilm, Tokyo, Japan) using Clarity Western ECL Substrate (Bio-Rad, Hercules, CA, USA). Antibodies used in this study were as follows: mouse monoclonal anti-Hsp105 (1:1000–2000; clone B-7, Santa Cruz Biotechnology, Dallas, TX, USA), anti-Hsp90 (1:2000; clone AC88, Enzo Life Sciences, Farmingdale, NY, USA), anti-Hsp70 (1:2000; clone C92F3A-5, Enzo Life Sciences) and rabbit monoclonal anti-*a*-tubulin (1:2000; clone DM1A, Sigma-Aldrich, St. Louis, MO, USA) antibodies. HRP-conjugated donkey anti-mouse (1:4000; 712-035-151, Jackson Immuno Research Laboratories Inc., West Grove, PA, USA) and donkey anti-rabbit (1:4000; 712-035-152, Jackson Immuno Research Laboratories Inc.) IgG antibodies.

### Statistical analysis

Statistical analysis was performed using GraphPad Prism 8.21 software. Statistical analysis was conducted using a one-way analysis of variance (ANOVA) followed by a Dunnett’s test or a Tukey–Kramer test to analyze the differences between the treatment groups. The significance level used for statistical analysis with two-tailed testing was 5%.

## Results and discussion

### Isolation of the compounds produced by *P. maximae* JKYM-AK1

The fungus, *P. maximae* JKYM-AK1 was cultured in a potato dextrose medium. The EtOAc soluble fraction was obtained from mycelia and supernatant after incubation of the culture at 28 °C for 20 days. The EtOAc soluble fraction was purified by normal and reversed phase silica gel column chromatography followed by HPLC to give four new compounds: maximazaphilones I–IV (**1–4**) together with three known compounds, isochromophilone IV (**5**) [28, 29], isochromophilone I (**6**) [30], and hypocrellone A (**7**) [31].

### Chemical structure of maximazaphilone I (1)

**Fig. 1:**
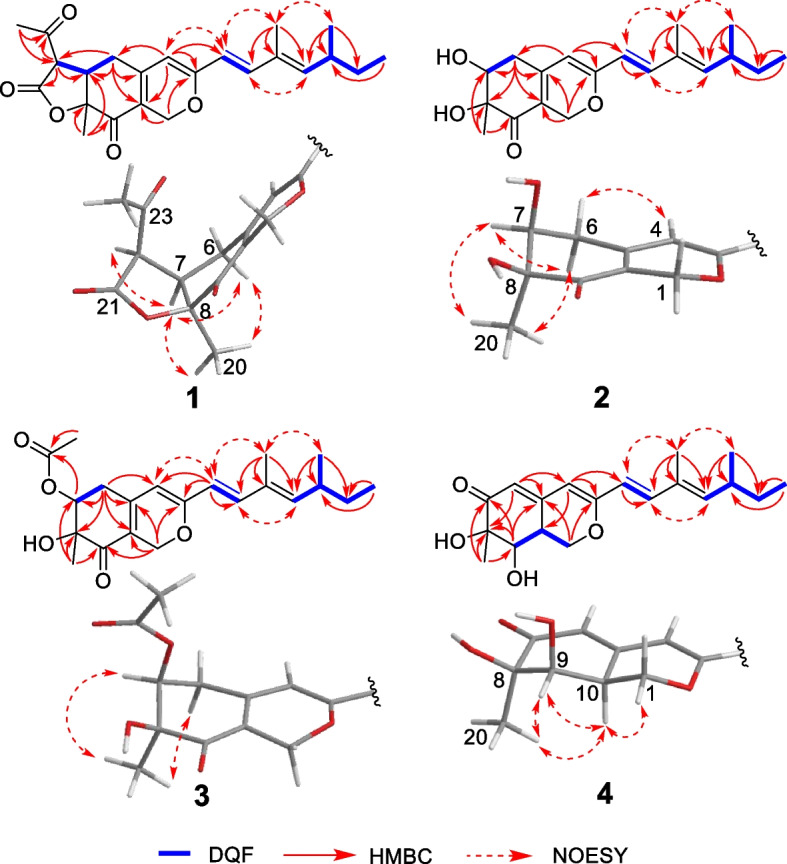
Important 2D NMR correlations of new maximazaphilones (**1**–**4**)

**Fig. 2:**
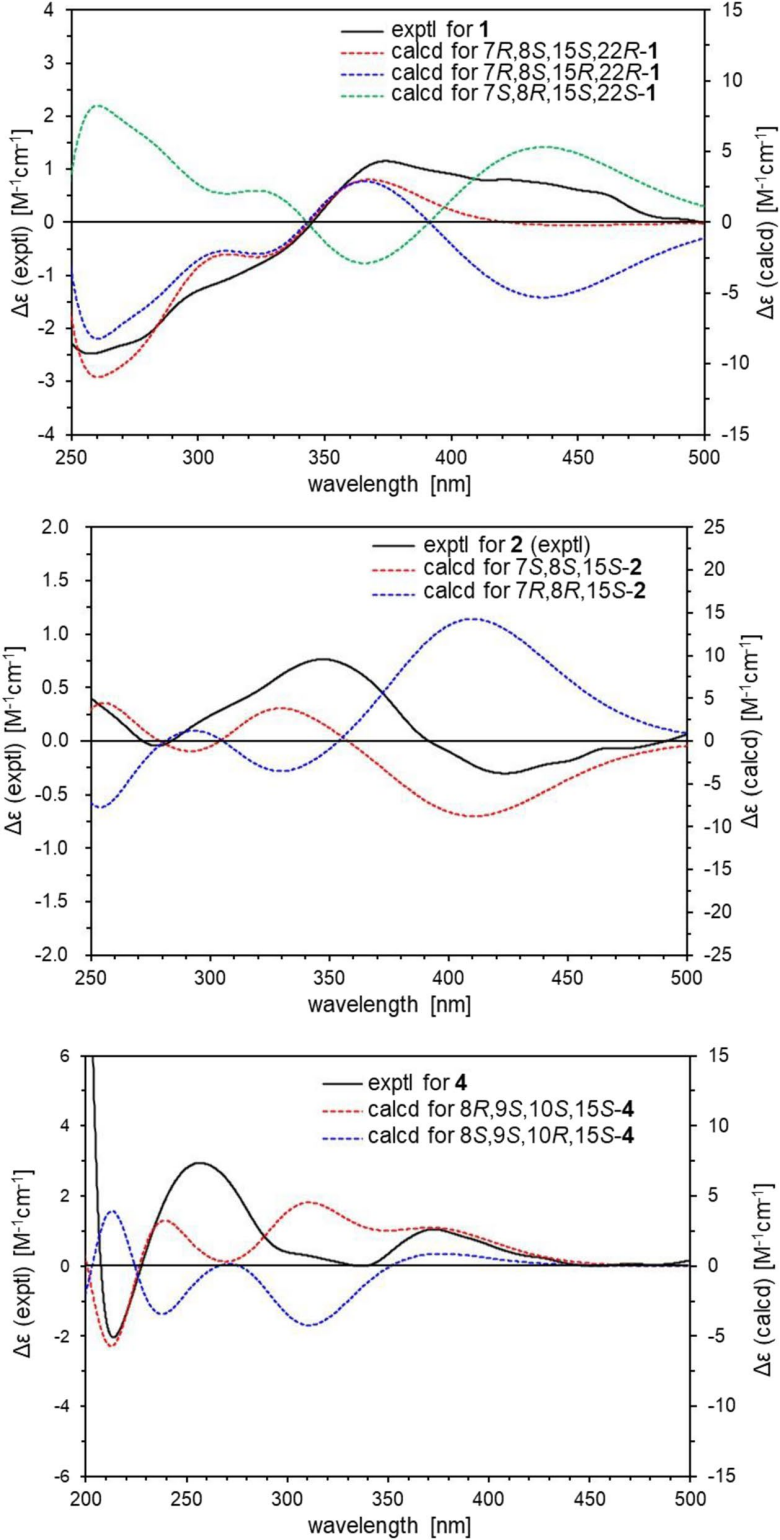
Comparison of the experimental and calculated ECD spectra for **1**, **2**, and **4**

**Fig. 3:**
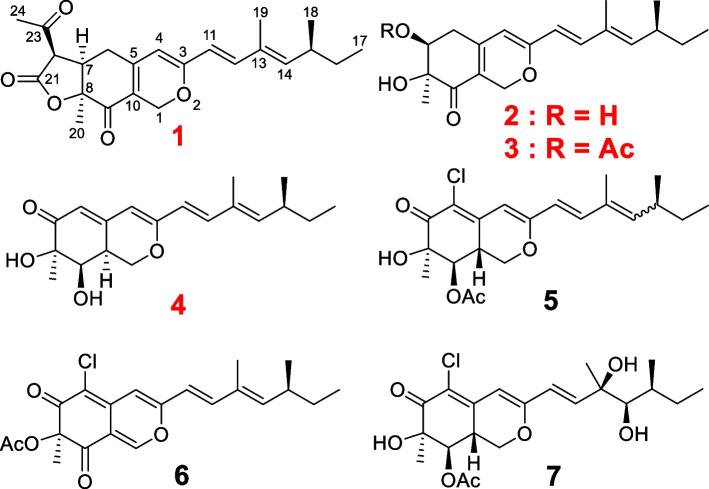
The chemical structures of the isolated azaphilones (**1**–**7**) produced by *P. maximae* JKYM-AK1

Maximazaphilone I (**1**) was isolated as a yellow amorphous powder with positive optical rotation ([*α*]^25^_D_ +44.2 in MeOH). Its molecular formula (C_23_H_28_O_5_) was determined using HRMS and ^13^ C NMR spectroscopy. A molecular ion peak was observed using FAB-MS for **1** (*m/z* 385 [M]^+^). The ^1^ H and ^13^ C NMR (CDCl_3_) spectra recorded for **1** (Table 1) were similar to those of monascusone B [32] except for the presence of side chain. Namely, the ^1^ H and ^13^ C NMR spectra showed characteristic signals for three carbonyl groups [*δ*_C_ 188.5 (C-9), 169.3 (C-21), and 200.3 (C-23)], two olefin groups [*δ*_C_ 162.7 (C-3), 104.3 (C-4), 144.0 (C-5), and 114.6 (C-10)], a methylene bearing oxygen function group [*δ*_Η_ 4.88 (d, *J* = 13.2, H-1) and 4.99 (d, *J* = 13.2, H-1)], a methylene group [*δ*_Η_ 2.43 (overlapping with other signal, H-6*b*) and 2.88 (dd, *J* = 6.6, 19.2, H-6)], two methines [*δ*_Η_ 3.26 (dd, *J* = 4.8, 12.6, H-7) and 3.59 (d, *J* = 12.6, H-22)], a quaternary carbon bearing oxygen function group [*δ*_C_ 82.7 (C-8)] and two methyl groups [*δ*_H_ 1.62 (s, H-20) and 2.46 (s, H-24)]. The positions of the side chain (C-11–C-19) was determined based on the DQF COSY and HMBC spectra shown in Fig. 1. Namely, the long-range correlations were observed between H-4/C-11 suggested the side chain was attached at C-3. The relative stereo structure except for C-15 and geometry of C-10–C-14 double bonds were determined by NOESY spectra. NOESY cross-peaks corresponding to H-6*a*/H-7, H-6*a*/H-20, H-7/H-20, and H-7/H-22 suggested that H-6*a*, H-7, H-22, and methyl group attached at C-8 were located at same side. NOESY cross-peaks corresponding to H-4/H-11, H-11/H-19, and H-12/H-14 established the geometry of C-11/12 and C-13/14 as *E* configurations. Finally, the absolute stereochemistry was determined by the calculated ECD spectra. The experimental ECD spectrum 250–400 nm for **1** was identical to that of calculated ECD spectra having 7*R*,8* S*,15* S*,22*R* and 7*R*,8* S*,15*R*,22*R* absolute stereochemistry. On the other hand, the calculated ECD spectra of 7* S*,8*R*,15* S*,22* S* were the opposite of the experimental data. Therefore, we concluded the absolute stereochemistry at the tricyclic ring is 7*R*,8* S*,22*R*. In addition, the calculated ECD spectrum for the diastereomer of **1** having 15*R* absolute stereochemistry at side chain showed negative cotton effect at 436 nm and **1** didn’t show this trend, therefore, the absolute stereo structure of C-15 was determined as *S* (Fig. 2). Based on all this evidence, the chemical structures of maximazaphilone I (**1**) was determined as shown in Fig. 3.

### Chemical structure of maximazaphilones II and III (2 and 3)

Maximazaphilones II and III (**2** and **3**) were isolated as a yellow amorphous powder with positive optical rotations (**2**, [*α*]^25^_D_ +100.3 in MeOH) (**3**, [*α*]^25^_D_ +54.4 in MeOH). The molecular formulas (**2**, C_19_H_26_O_4_) and (**3**, C_21_H_28_O_5_) were determined using HRMS and ^13^ C NMR spectroscopy. The ^1^ H and ^13^ C NMR (CDCl_3_) spectra recorded for **2** (Table 1) show signals very similar to that of asperpyranone [33], however, the absolute stereochemistry of C-7 has not been described. A comparative study of **2** with maximazaphilone III (**3**) indicated the presence of an additional acetyl group also proved by its molecular formula and ^13^ C NMR spectra [**3**, *δ*_C_ (ppm) 170.6 (O*C*OCH_3_) and 21.1 (O*C*OCH_3_)]. The acetyl group in **3** is present at C-7 as established from the HMBC correlation between H-7/O*C*OCH_3_. NOESY cross peaks of **2** and **3** corresponding to [**2**, H-6*a*/H-7 and H-7/H-20] and [**3**, H-6*a*/H-20 and H-7/H-20] suggested that H-6*a*, H-7, and H-20 are located at the same side (Fig. 1). Therefore, the relative structures of **2** and **3** were determined as 7 S* and 8 S*. The absolute stereochemistry of C-15 was assumed to be *S* similar to **1** and other known azaphilones. The experimental ECD spectra of **2** and **3** were identical to that of the calculated ECD spectra having 7* S*,8* S*,15* S* absolute stereochemistry. Conversely, the calculated ECD spectrum of *ent*-**2** (7*R*,8*R*,15*R*) was opposite of the experimental data (Fig. 2). The absolute stereochemistry for **3** was deduced to be same as **2**. Therefore, the absolute stereochemistry for both **2** and **3** was determined as 7* S*,8* S*,15* S*. Based on these data, the determined chemical structures of maximazaphilone II and III (**2** and **3**) are shown in Fig. 3.

**Table 1:** ^13^ C NMR (150 MHz) and ^1^ H NMR spectroscopic data (600 MHz) of **1**–**4** in CDCl_3_

Position	1		Position	2		3		4	
	*δ*C	δH *(J* in Hz)		*δ*C	*δ*H *(J* in Hz)	*δ*C	*δ*H *(J* in Hz)	*δ*C	*δ*H *(J* in Hz)
1	63.7	4.88 (d, *J* = 13.2) 4.99 (d, *J* = 13.2)	1	63.9	4.80 (d, *J* = 12.6) 5.06 (d, *J* = 12.6)	63.9	*a* 4.86 (d, *J* = 13.2) *b* 5.04 (d, *J* = 13.2)	68.2	*a* 4.49 (dd, *J* = 4.8, 11.4) *b* 4.22 (m)
3	162.7		3	161.9		161.9		161.0	
4	104.3	5.31 (s)	4	105.0	5.37 (s)	104.4	5.33 (s)	104.8	5.70 (s)
5	149.0		5	149.0		147.8		151.6	
6	26.4	*a* 2.88 (dd, *J* = 6.6, 19.2) *b* 2.43 (overlapped)	6	32.5	*a* 2.75 (m) *b* 2.69 (m)	32.0	*a* 2.84 (d-like, *J* = 19.2) *b* 2.63 (d-like, *J* = 19.2)	113.2	5.78 (s)
7	42.1	3.26 (dd, *J* = 4.8, 12.6)	7	73.0	4.16 (m)	74.9	5.33 (overlapped)	199.4	
8	82.7		8	75.6		74.0		76.4	
9	188.5		9	195.9		195.2		74.5	4.14 (d, *J* = 2.4)
10	114.6		10	112.2		112.8		36.6	3.00 (m)
11	117.6	5.86 (d, *J* = 15.6)	11	118.1	5.89 (d, *J* = 15.0)	117.9	5.89 (d, *J* = 15.6)	118.9	5.89 (d, *J* = 16.2)
12	142.6	6.96 (d, *J* = 15.6)	12	141.1	6.93 (d, *J* = 15.0)	141.3	6.95 (d, *J* = 15.6)	140.6	6.92 (d, *J* = 16.2)
13	132.1		13	132.1		132.1		132.1	
14	147.6	5.61 (d, *J* = 9.6)	14	146.6	5.58 (d, *J* = 9.6)	146.7	5.59 (d, *J* = 10.2)	146.1	5.57 (d, *J* = 9.6)
15	35.0	2.43 (overlapped)	15	34.9	2.44 (m)	35.0	2.44 (m)	34.9	2.44 (m)
16	30.1	1.28 (m) 1.40 (m)	16	30.1	1.29 (m) 1.40 (m)	30.1	1.29 (m) 1.39 (m)	30.2	1.30 (m) 1.41 (m)
17	11.9	0.83 (t, *J* = 7.8)	17	11.9	0.83 (t, *J* = 7.8)	11.9	0.84 (t, *J* = 7.2)	11.9	0.85 (t, *J* = 7.2)
18	20.3	0.97 (d, *J* = 7.8)	18	20.3	0.98 (d, *J* = 6.0)	20.3	0.98 (d, *J* = 6.6)	20.4	0.98 (d, *J* = 6.0)
19	12.4	1.77 (s)	19	12.4	1.79 (s)	12.4	1.79 (s)	12.4	1.79 (s)
20	21.7	1.62 (s)	20	23.5	1.33 (s)	24.2	1.39 (s)	23.3	1.36 (s)
21	169.3		*C*OCH_3_			170.6			
22	56.6	3.59 (d, *J* = 12.6)	CO*CH*_*3*_			21.1	2.02 (s)		
23	200.3								
24	30.0	2.46 (s)							

### Chemical structure of maximazaphilone IV (4)

Maximazaphilone IV (**4**) was isolated as a yellow amorphous powder with a positive optical rotation ([*α*]^25^_D_ +173.0 in MeOH). The molecular formula of **4** (C_19_H_26_O_4_) was determined using HRMS and ^13^ C NMR spectroscopy. The ^1^ H and ^13^ C NMR (CDCl_3_) spectra recorded for **4** (Table 1) show signals close to those of dechloroisochromophilone [34] and (8* S*,9* S*,10* S*)-dechloroisochromophilone [35] except for the signals corresponding to C-7 [*δ*_C_ 199.4], C-8 [*δ*_C_ 76.4], C-9 [*δ*_C_ 74.5], and C-10 [*δ*_C_ 36.6]. These findings suggested that **4** should be the diastereomer of these two known compounds. NOESY cross-peaks of **4** corresponding to H-20/H-9, H-20/H-10, H-9/H-10 suggested that H-20, H-9, and H-10 were located at same side (Fig. 1). Therefore, the relative structure of **4** were determined as 8R*, 9R*, and 10 S*. The absolute stereochemistry of **4** was determined by the calculated ECD spectra and was found to be the same as **1–3** (Fig. 2). Based on these data, the chemical structure of maximazaphilone IV (**4**) was determined as shown in Fig. 3.

### Evaluation of the inhibitory effects of isolated compounds against Hsp105 expression using luciferase assay system

**Fig. 4:**
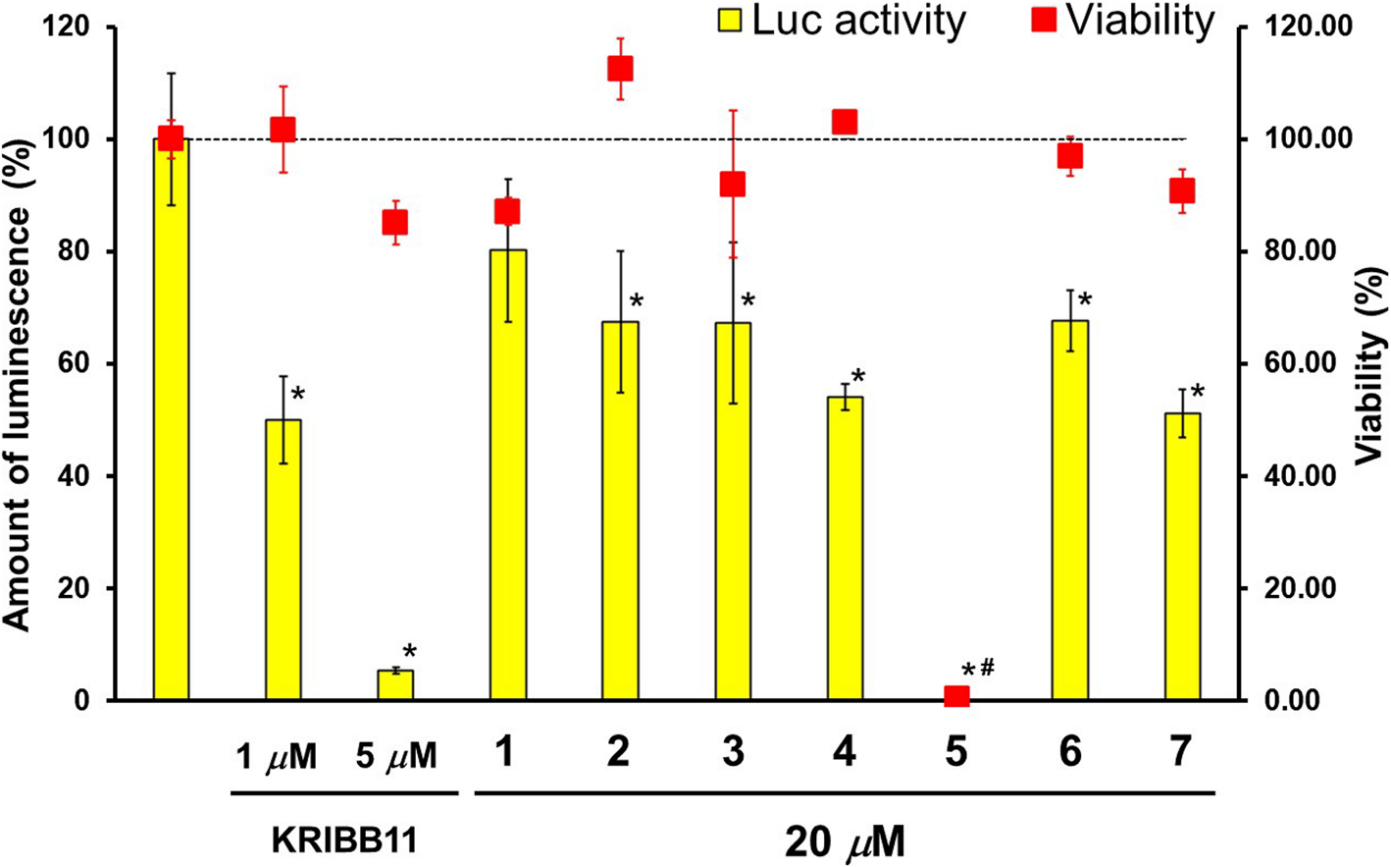
The inhibitory effects of the isolated compounds (**1**–**7**) against heat shock promoter activity using a luciferase assay system with pGL105/C3H cells. KRIBB11 was used for positive control. The percentages of luciferase activities and cell viabilities were described as means ± SD (*n* = 3) from one of the three independent experiments. Statistical significance was analyzed using the Dunnett’s test (*P < 0.01, #*P* < 0.01 compared with each control group)

Inhibitory effects of the isolated compounds (**1**–**7**) against the expression of Hsp105 were evaluated for heat shock promoter activity using a luciferase (*luc*) assay system using pGL105/C3H cells. pGL105/C3H cells were mouse C3H10T1/2 cells stably transfected with pGL105 reporter plasmid containing the Hsp105 promoter upstream of a luciferase gene. In this assay, the inhibitory effect of test samples was assessed by observing the decrease of Luc activity [26]. KRIBB11 was used as a positive control. KRIBB11 had been reported to be an inhibitor of the heat shock factor 1, the transcription factor of several Hsps including Hsp105 [3]. **2**–**7** significantly decreased Luc activity; however, **5** significantly inhibited cell proliferation (Fig. 4). These results suggested that **2–4**, **6**, and **7** should have inhibitory effects against Hsp105 expression on pGL105/C3H cells. We could not determine whether the inhibitory effect against Luc activity is caused by cytotoxicity in the case of **5**.

### Evaluation of the cell death inducing activity on ADR-treated HeLa cells

**Fig. 5:**
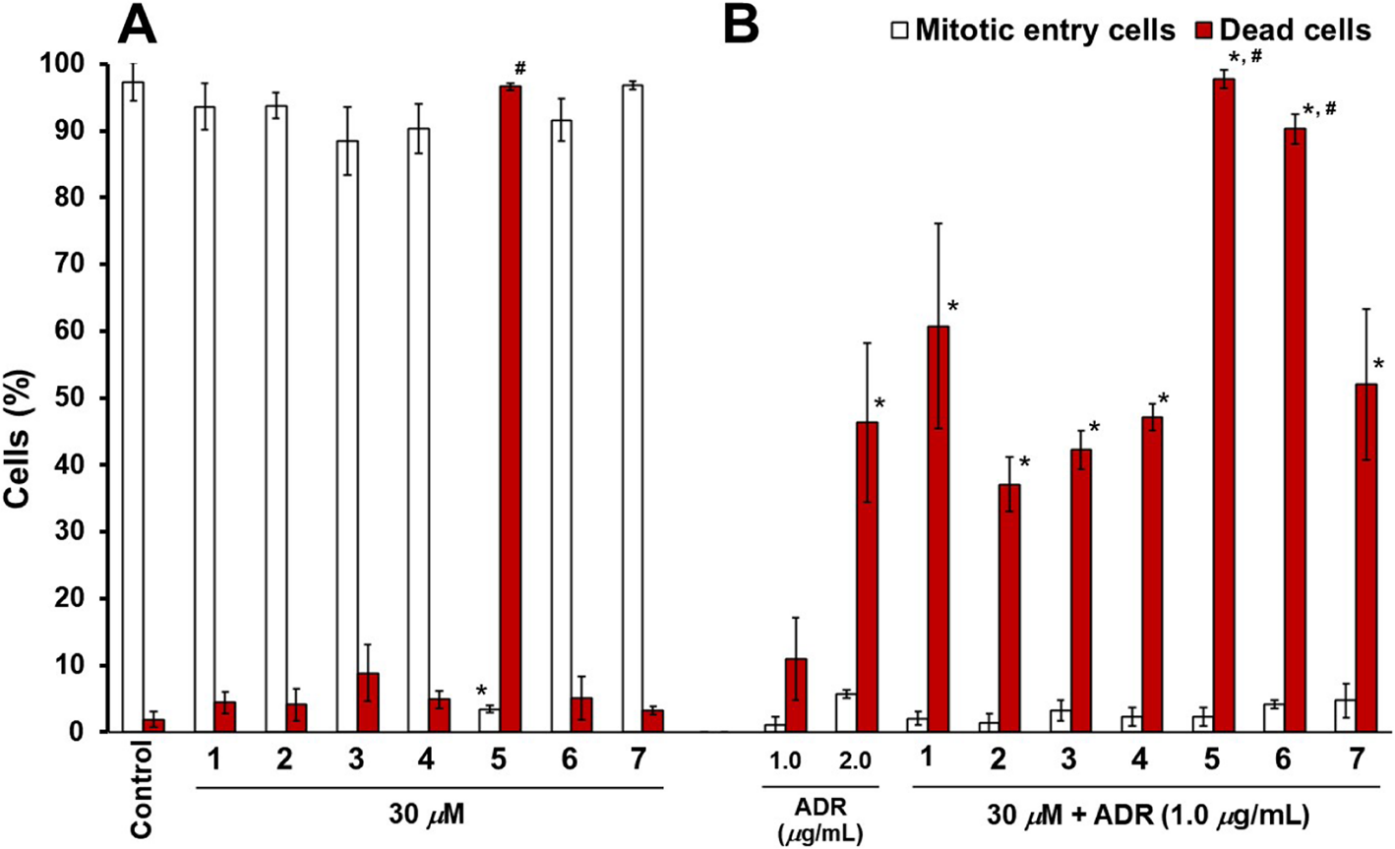
Effects of the isolated compounds (**1** − **7**) on cell proliferation and death. The number of mitotic entry cells and dead cells were counted during time-lapse imaging. The percentages of mitotic entry cells or dead cells are reported as means ± SD of three different visual fields. Each field captured more than 100 cells. **A** HeLa cells were treated with 30 *µ*M indicated compounds. Statistical significance was analyzed using the Tukey-Kramer test (*P < 0.01 compared with control group). **B** ADR (1.0 *m*g/ml) or combination of ADR (1.0 *m*g/ml) and isolated compounds (**1** − **7**) for 24 h [*P < 0.01 compared with ADR (1.0 *m*g/ml) group. ^*#*^ *P* < 0.01 compared with ADR (2.0 *m*g/ml) group

Hsp105, a molecular chaperone, suppresses ADR-induced cell death via anti-apoptotic functions [9]. Our previous study suggested that the cell cycle arrest was induced via DNA damage, however, low concentrations of ADR (0.1 − 1.0 *µ*g/ml) do not induce cell death [10]. Therefore, the compounds that inhibit the functions or expression of Hsp105 should increase the number of dead cells on low concentration ADR treatment cells. We evaluated the cell-death inducing activities of isolated compounds on HeLa cells for 24 h using time-lapse imaging analysis. In this study, we counted the number of mitotic entry cells and dead cells under treatment of test compounds (**1** − **7**) (30 *µ*M) (Fig. 5A), ADR (1.0 and 2.0 *µ*g/ml), and combination of test compounds (30 *µ*M) with ADR (1.0 *µ*g/ml) (Fig. 5B). Treatment of **1**–**4**, **6**, and **7** did not affect the number of dead cells and mitotic entry cells. On the other hand, **5** increased the number of dead cells and decreased the number of mitotic entry cells. The combination treatment of all isolated compounds (**1**–**7**) with ADR (1.0 *µ*g/ml) significantly increased the dead cells compared to those of ADR-treated cells (1.0 *µ*g/ml). Therefore, **1**–**4**, **6**, and **7** may suppress the anti-apoptotic functions of HeLa cells. Moreover, the number of dead cells by combination treatment of **6** with ADR (1.0 *µ*g/ml) was significantly larger than that of ADR (2.0 *µ*g/ml) treatment group. We also showed that HSF1 inhibitor KRIBB11 (positive control) increased ADR sensitivity (Fig. S4) and dose dependent cell death-inducing activity (Fig. S 5). These results suggest that **6** may be able to reduce the dose of ADR less than 50% to avoid the side effects.

According to the results of luciferase assay and cell death-inducing activity, **6** may have the inhibitory effects against Hsp105 expression. Therefore, we evaluated the expression of Hsp105 and major Hsps, Hsp90 and Hsp70, in HeLa cells treated with **6** using western blotting analysis. Treatment with 100 *µ*M **6** inhibited the expression of Hsp105 and Hsp90 under heat shock conditions (Fig. S6), however, 30*µ*M **6** didn’t affect the expression of these Hsps (data not shown). Therefore, we concluded that the mechanisms of cell death inducing activity for **6** may be not only inhibition of the expression for Hsp105 and Hsp90 but also other effects that contributing to drug resistance.

## Conclusion

We isolated new azaphilone, named maximazaphilones I–IV (**1–4**) together with known compounds (**5**–**7**) from the mycelia and the culture supernatant of *P. maximae* JKYM-AK1. Among the isolated compounds, **2**–**4**, **6**, and **7** were suggested to have inhibitory effects against Hsp105 expression on luciferase reporter assay using pGL105/C3H cells. The combination treatment of all isolated compounds with ADR (1.0 *µ*g/ml) significantly increased the dead cells compared to those of ADR-treated cells (1.0 *µ*g/ml). The previous report suggested that some azaphilones have the inhibitory effects against Hsp90 via interacting with the N-terminal domain [8], therefore, the mechanism of cell death inducing activity for **2**–**4**, **6**, and **7** against ADR-treated Hela cells may be not only inhibition of Hsp90 but also inhibition of the expression for Hsp105. Based on this evidence, we concluded that the azaphilone **2**–**4**, **6**, and **7** have a potency for cancer treatment to reduce the dose of anticancer agents via inhibition of Hsps expression including Hsp105.

## Supplementary Information

**Additional file 1: S1. **^1^H NMR spectra of maximazaphilones I–IV (**1**–**4**). **S2.** ^13^C NMR spectra of maximazaphilones I–IV (**1**–**4**). **S3.** TheDFT-optimized structures of conformers of maximazaphilones I, II, and IV (**1**, **2**, and **4**) with their equilibrium population. **S4.** Effects of the KRIBB11 (positive control) on cell proliferation and death. **S5.** Effects of the compounds **6 **and **7** at two concentrations (10 *μ*M and 30 *μ*M) on cell proliferation and death. **S6. **The expression of HSP on HeLa cell treated with **6**.

## Data Availability

Not applicable.
