# Supplementary material for: Azaphilones produced by Penicillium maximae with their cell death-inducing activity on Adriamycin-treated cancer cell
Source: Genes Environ. 2023 Jan 19;45:5. doi: 10.1186/s41021-023-00261-w (PMC9850696; doi:10.1186/s41021-023-00261-w)
Supplement: Supplementary file 1 — Additional file 1: S1. 1H NMR spectra of maximazaphilones I–IV (1–4). S2. 13C NMR spectra of maximazaphilones I–IV (1–4). S3. TheDFT-optimized structures of conformers of maximazaphilones I, II, and IV (1, 2, and 4) with their equilibrium population. S4. Effects of the KRIBB11 (positive control) on cell proliferation and death. S5. Effects of the compounds 6 and 7 at two concentrations (10 μM and 30 μM) on cell proliferation and death. S6. The expression of HSP on HeLa cell treated with 6. [file 41021_2023_261_MOESM1_ESM.docx]

Azaphilones produced by *Penicillium maximae* with their cell death-inducing activity on Adriamycin-treated cancer cell

Takahiro Matsumoto^1*^・Erika Ohnishi^1^・Takahiro Kitagawa^1^・Masaya Okayama^1^・Youhei Saito^1^・Hayato Yoshikawa^1^・Tomoe Ohta^2^・Tatsusada Yoshida^2^・Yuji Nakayama^1^・Tetsushi Watanabe^1*^

^1^ Kyoto Pharmaceutical University, 1 Misasagi-Shichono-cho, Yamashina-ku, Kyoto 607-8412, Japan.

^2^ Faculty of Pharmaceutical Sciences, Nagasaki International University, 2825-7 Huis Ten Bosch-Cho, Sasebo, Nagasaki 859-3298, Japan.

* Corresponding authors. Phone: +81 75 595 4650 Fax: +81 75 595 4769 E-mail: [tmatsumo@mb.kyoto-phu.ac.jp](mailto:tmatsumo@mb.kyoto-phu.ac.jp) (T.M.), [watanabe@mb.kyoto-phu.ac.jp](mailto:watanabe@mb.kyoto-phu.ac.jp) (T.W.)

**List of Supporting Information**

1. ^1^H NMR spectra of maximazaphilones I–IV (**1**–**4**).
2. ^13^C NMR spectra of maximazaphilones I–IV (**1**–**4**).
3. The DFT-optimized structures of conformers of maximazaphilones I, II, and IV (**1**, **2**, and **4**) with their equilibrium population.
4. Effects of the KRIBB11 (positive control) on cell proliferation and death.
5. Effects of the compounds **6** and **7** at two concentrations (10 μM and 30 μM) on cell proliferation and death.
6. The expression of HSP on HeLa cell treated with **6**.


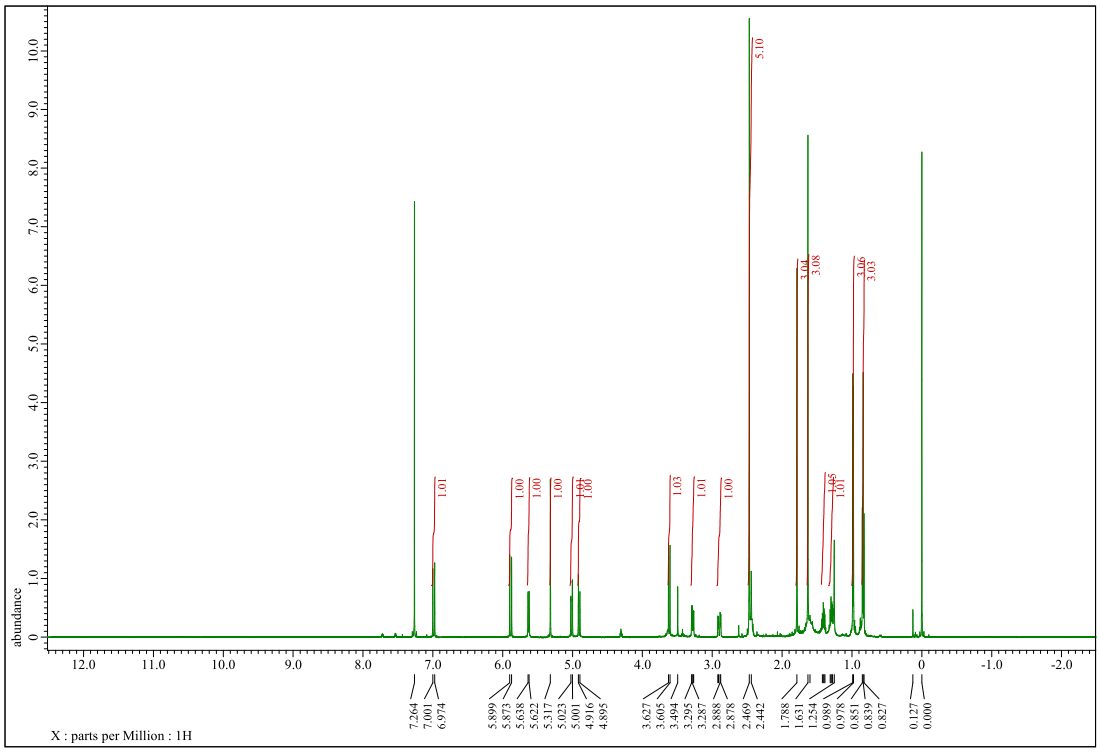
S1. ^1^H NMR spectra of maximazaphilones I–IV (1–4).


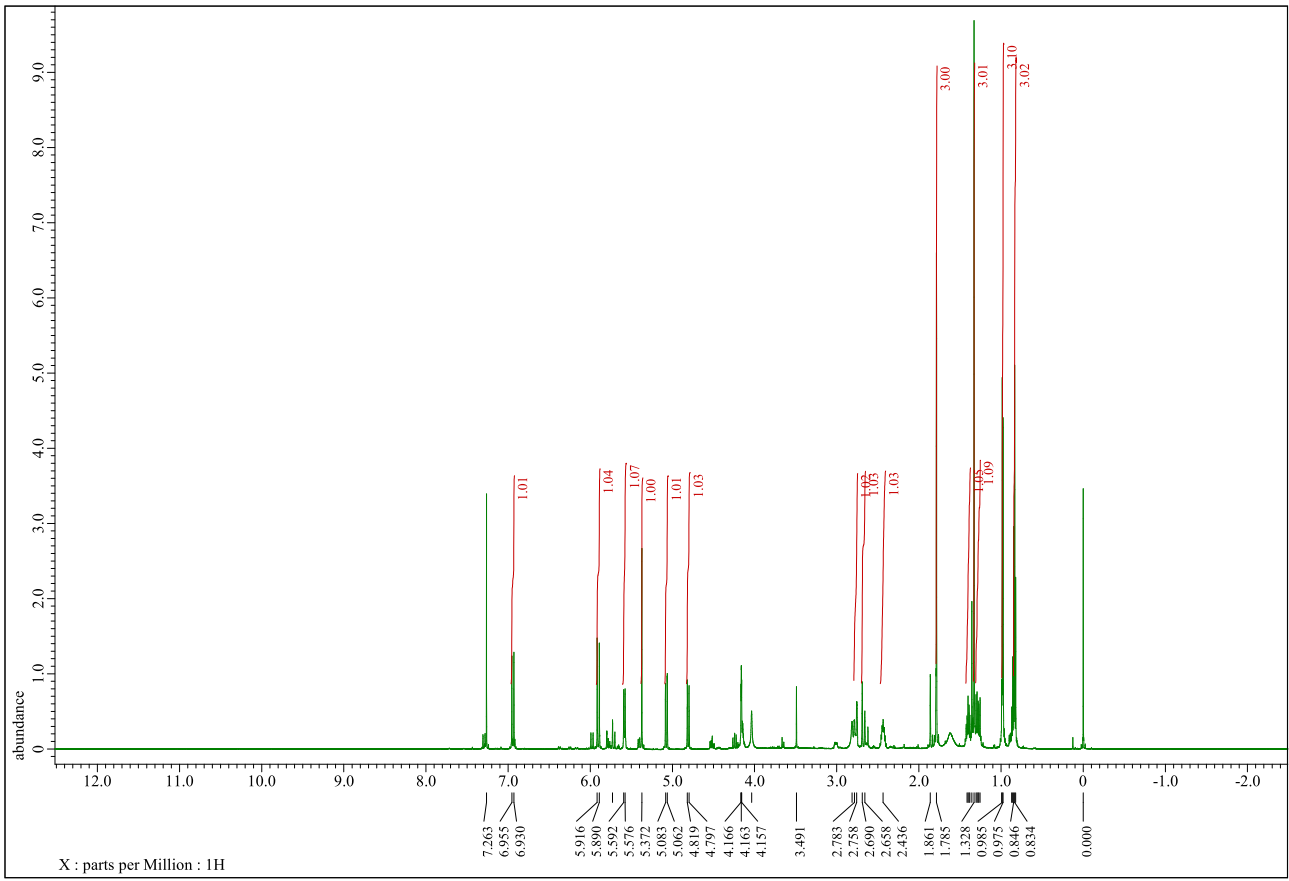
Fig. S1-1 ^1^H NMR spectra of maximazaphilone I (**1**)

Fig. S1-2 ^1^H NMR spectra of maximazaphilone II (2)


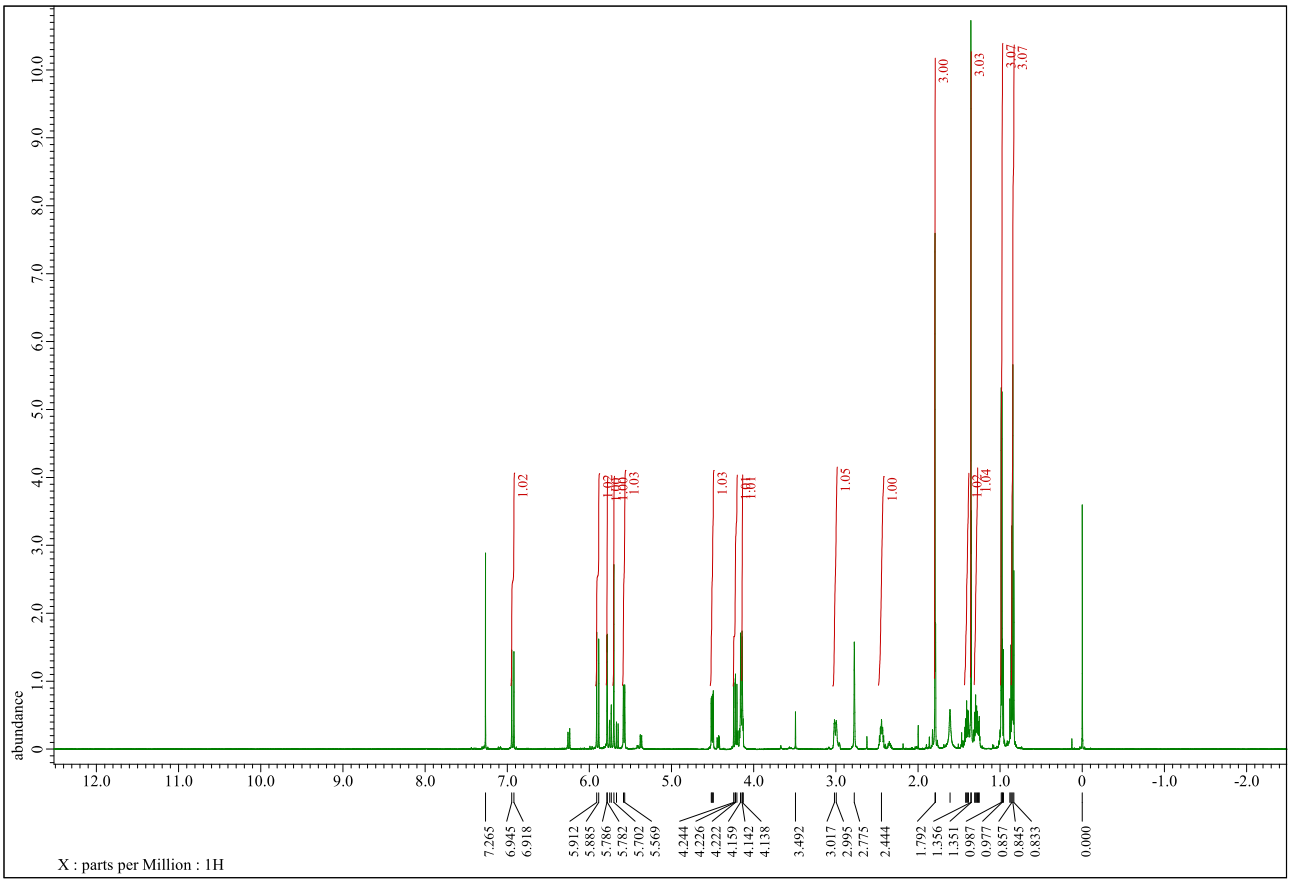

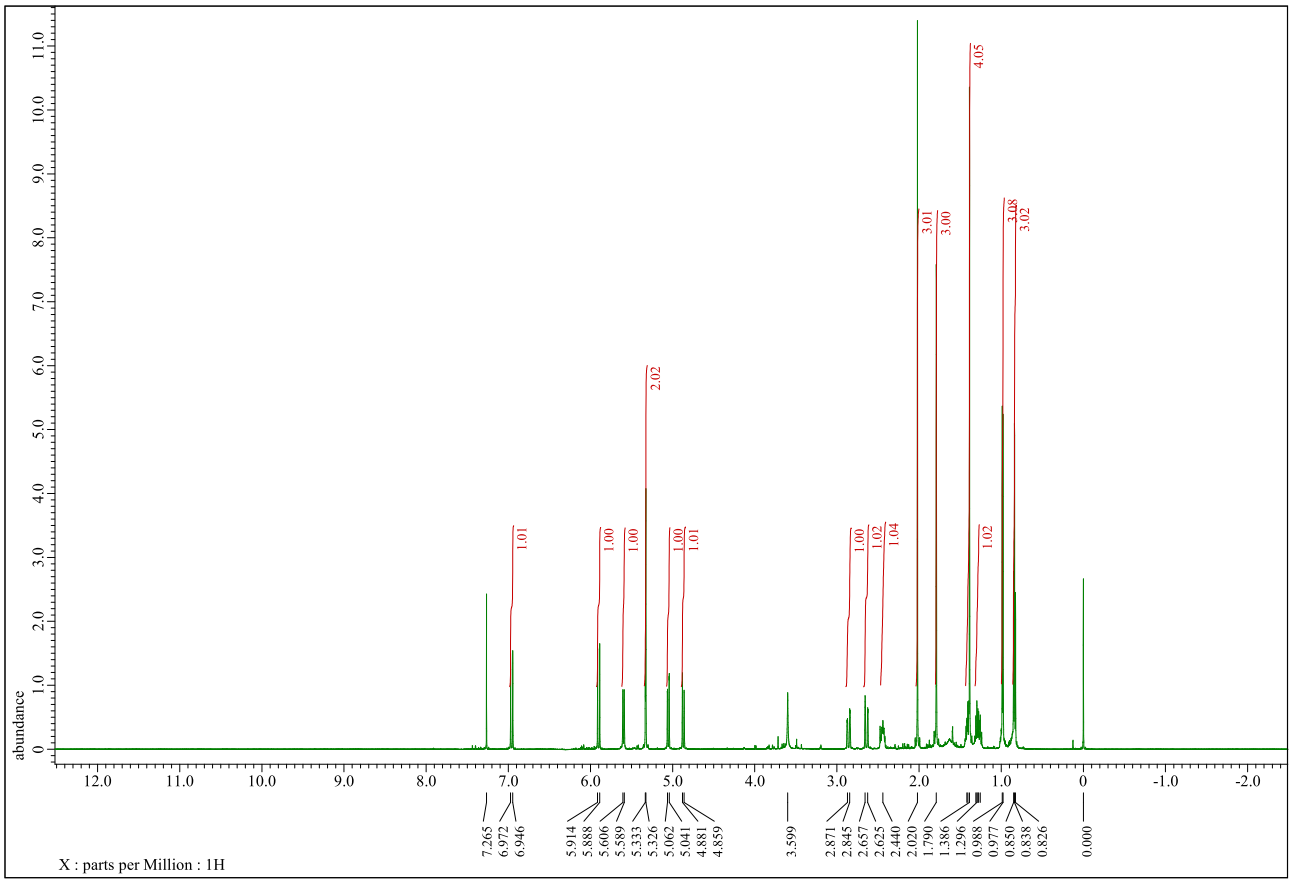
Fig. S1-3 ^1^H NMR spectra of maximazaphilone III (3)

Fig. S1-4 ^1^H NMR spectra of maximazaphilone IV (4)


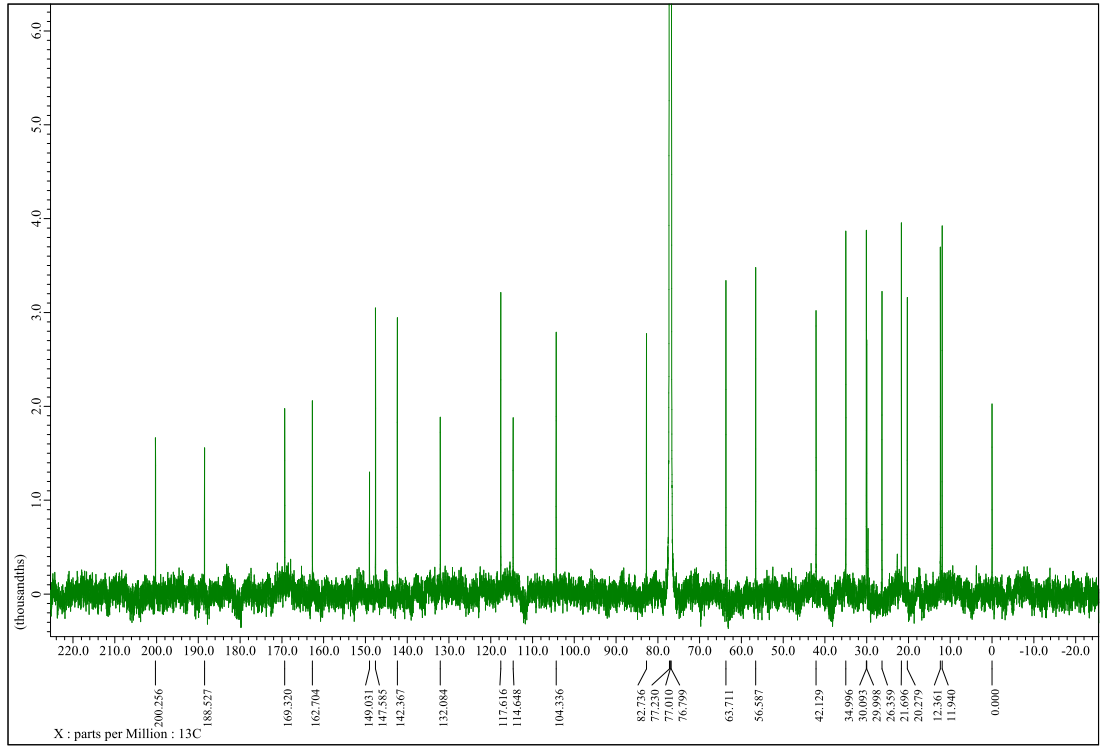
**
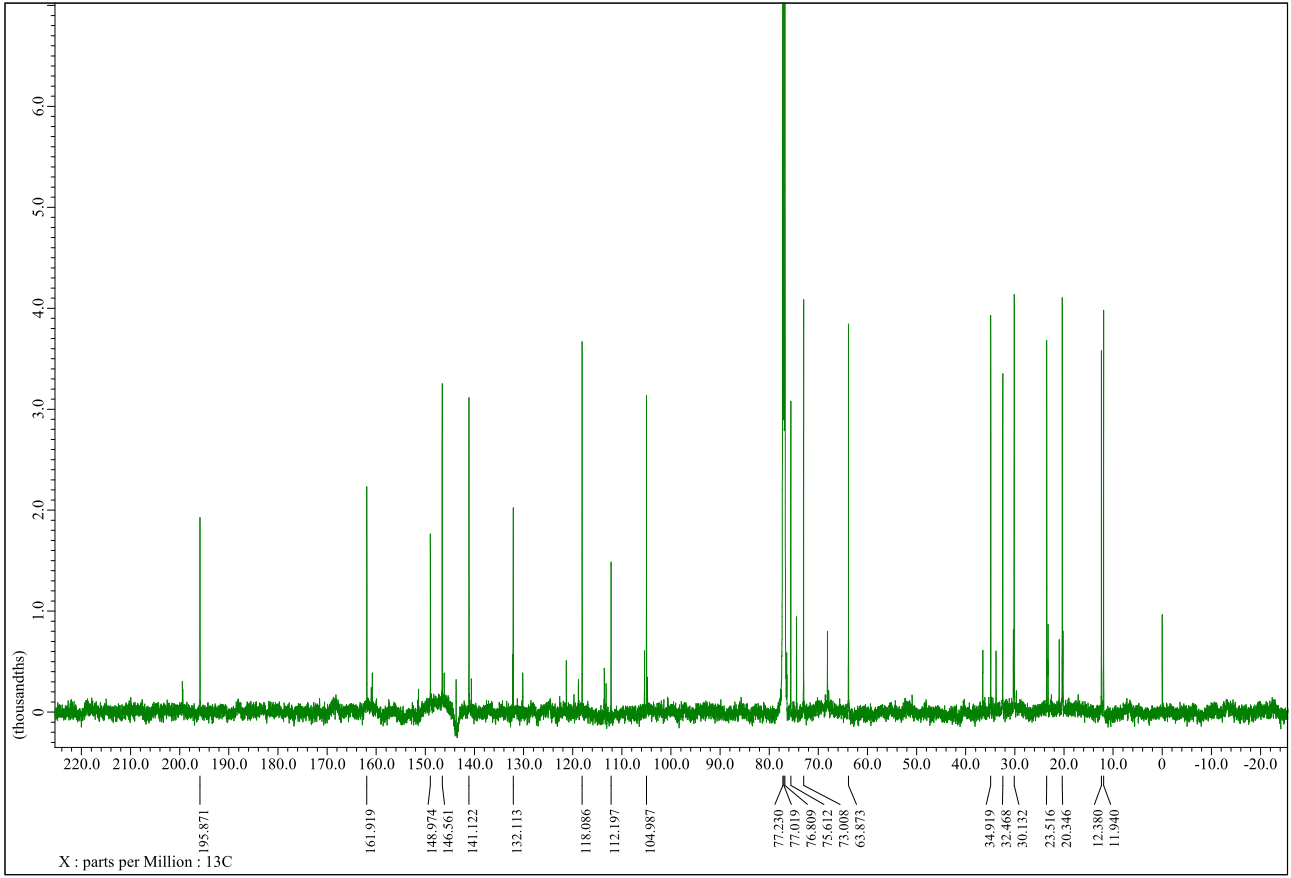
**S2. ^13^C NMR spectra of maximazaphilones I–IV (**1**–**4**).

Fig. S2-1 ^13^C NMR spectra of maximazaphilone I (**1**)

Fig. S2-2 ^13^C NMR spectra of maximazaphilone II (**2**)


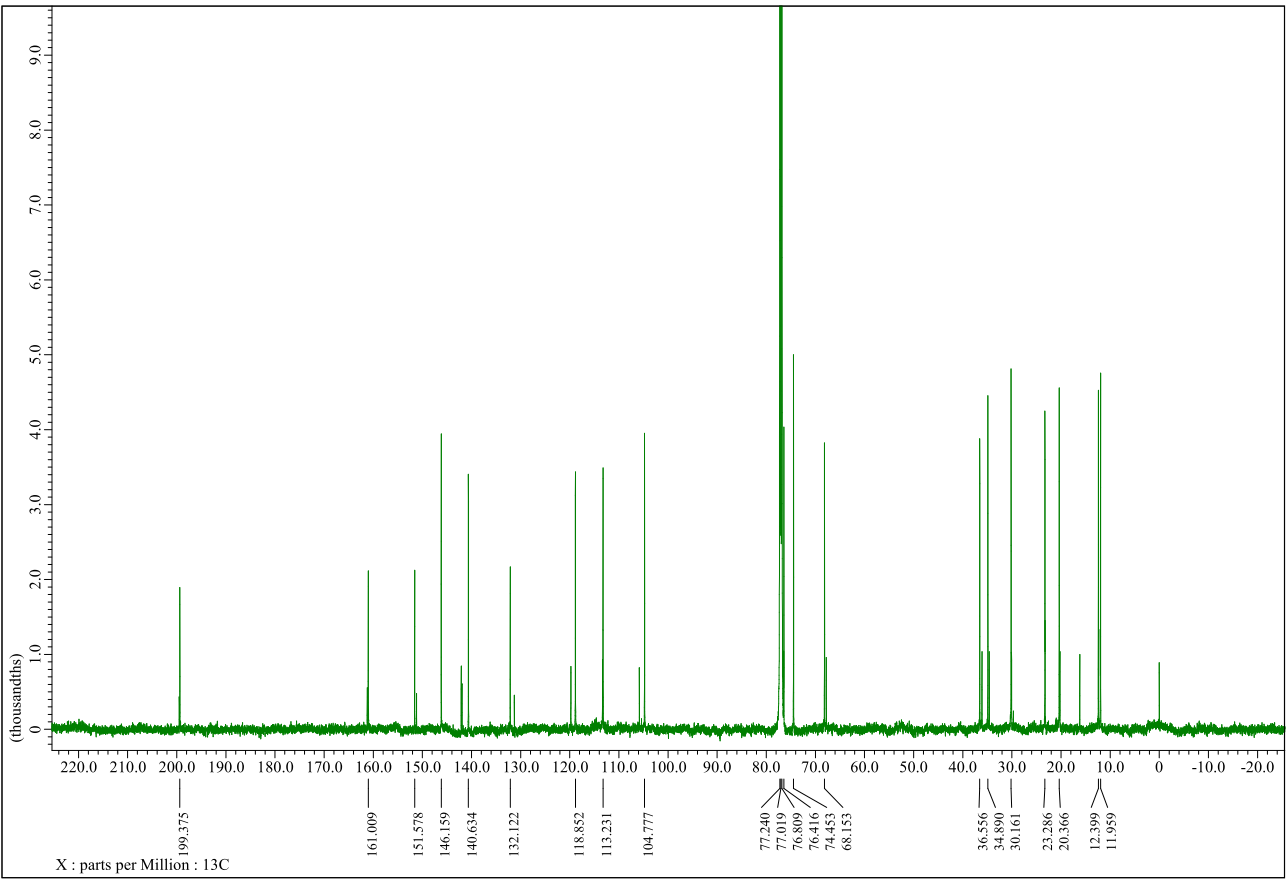
Fig. S2-
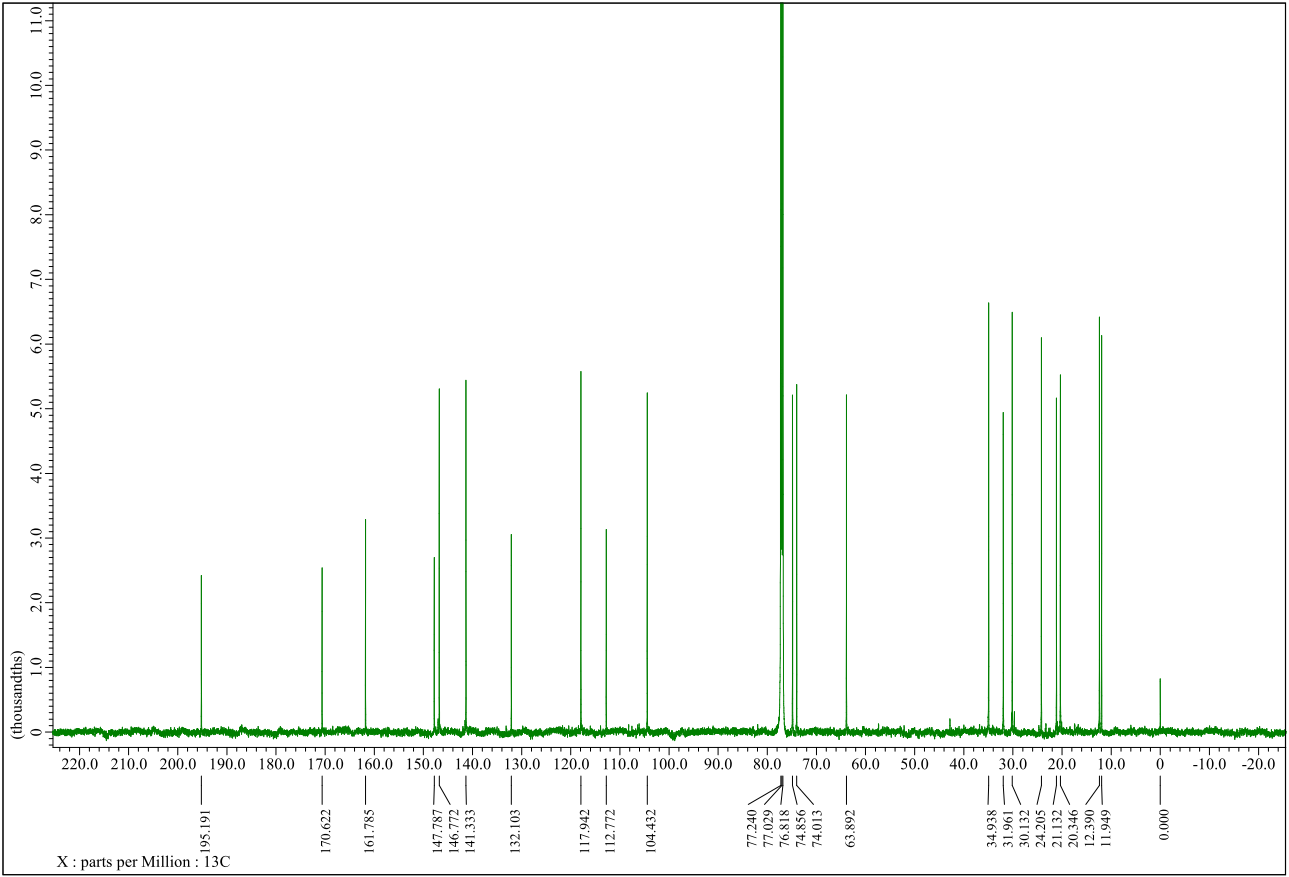
3 ^13^C NMR spectra of maximazaphilone III (**3**)Fig. S2-4 ^13^C NMR spectra of maximazaphilone IV (**4**)

S3. Optimized geometries, the minimum value of frequency, relative free-energies, and Boltzmann distributions of maximazaphilones I, II, and IV (1, 2, and 4).


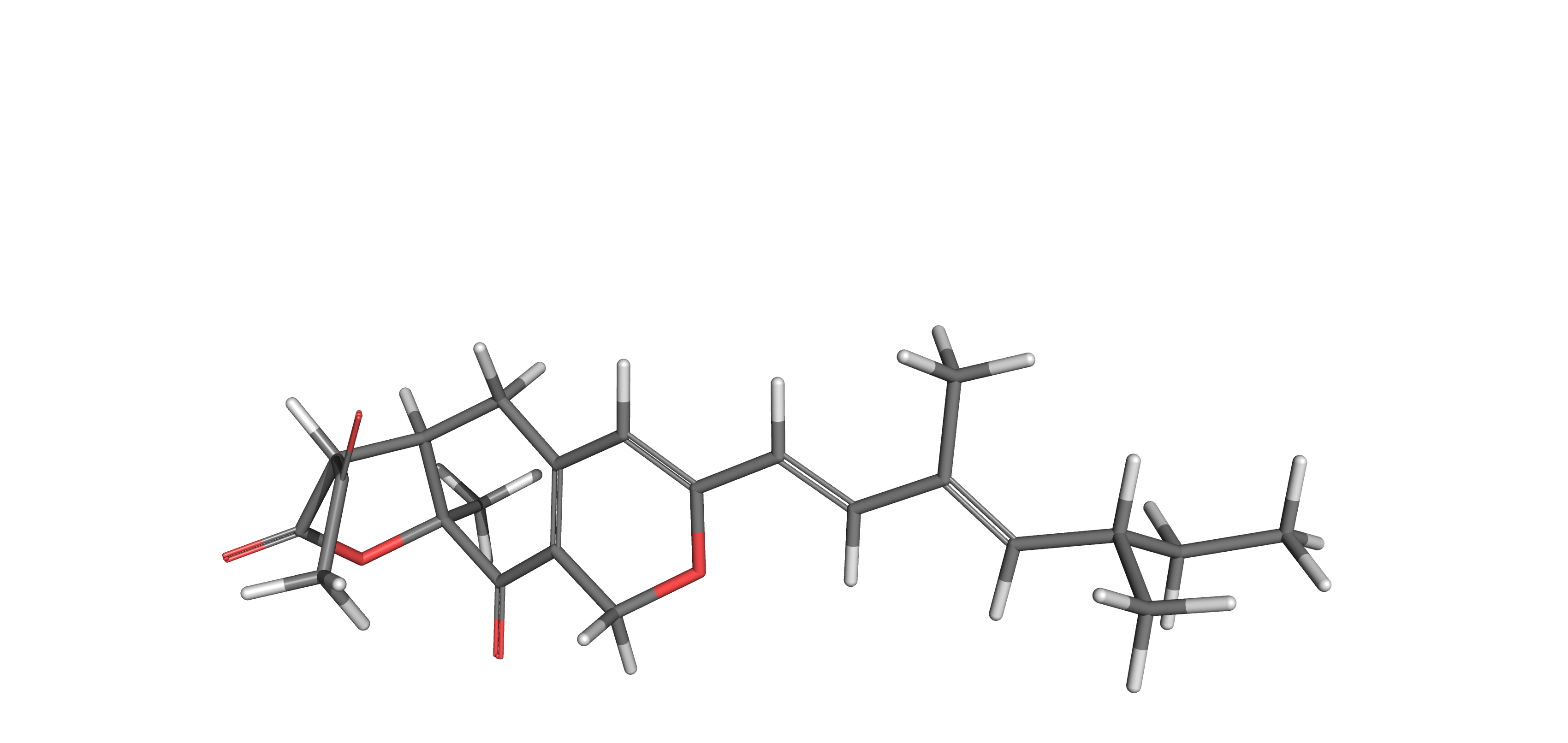

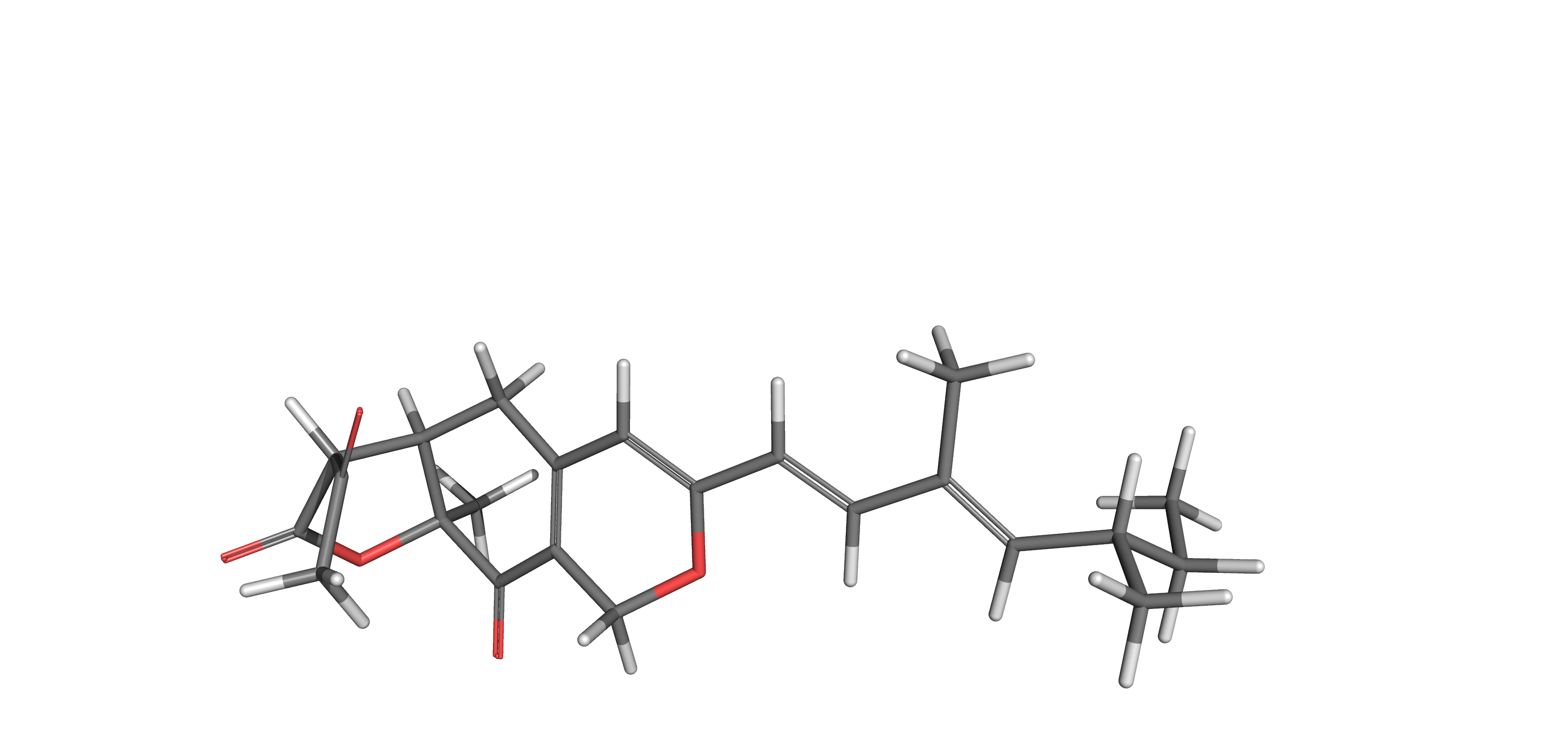


**Conf. 2**

[17.57]

Δ*G* = 0.66

P = 20.72

**Conf. 1**

[16.10]

Δ*G* = 0.00

P = 62.59


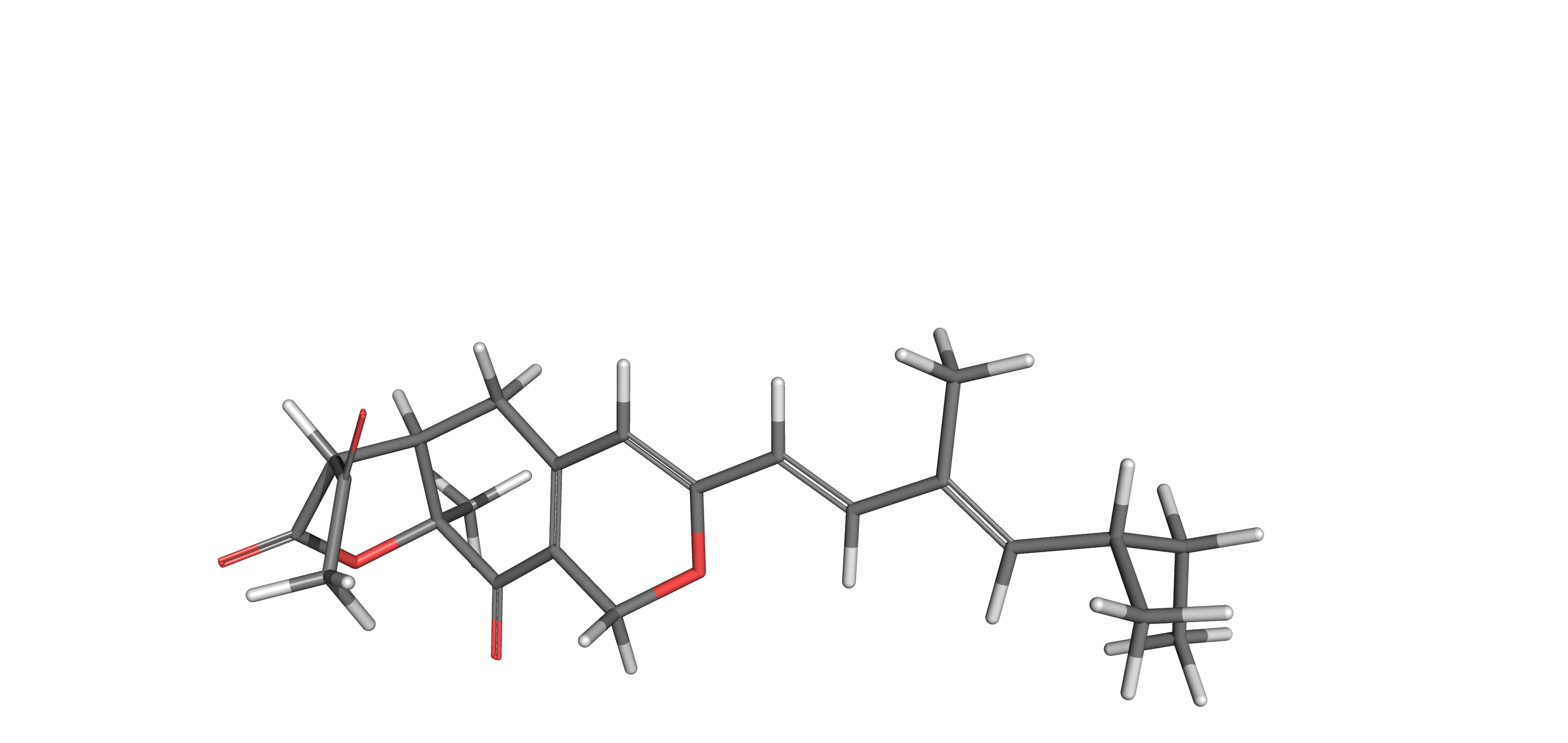

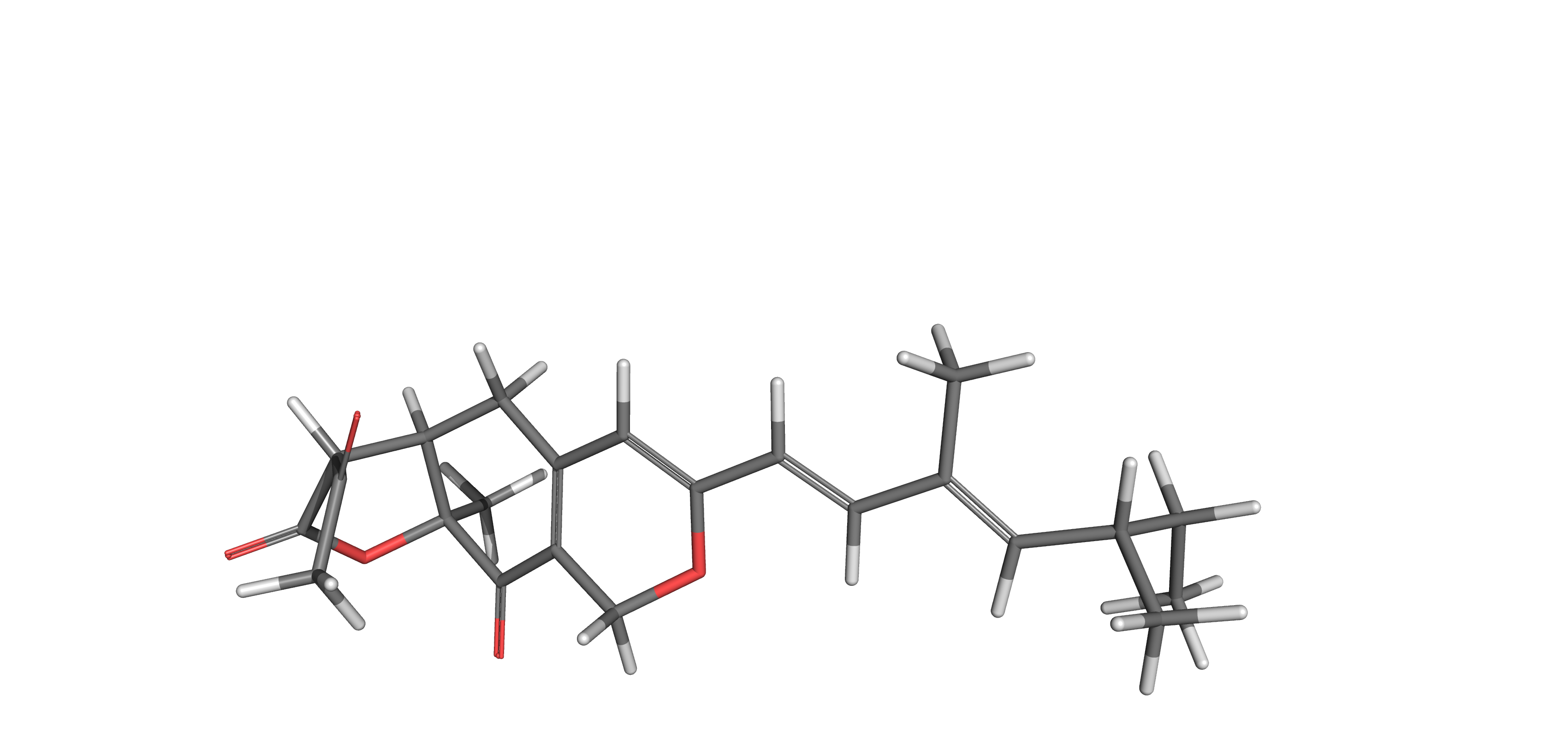


**Conf. 4**

[15.74]

Δ*G* = 1.54

P = 4.64

**Conf. 3**

[15.83]

Δ*G* = 1.45

P = 5.45


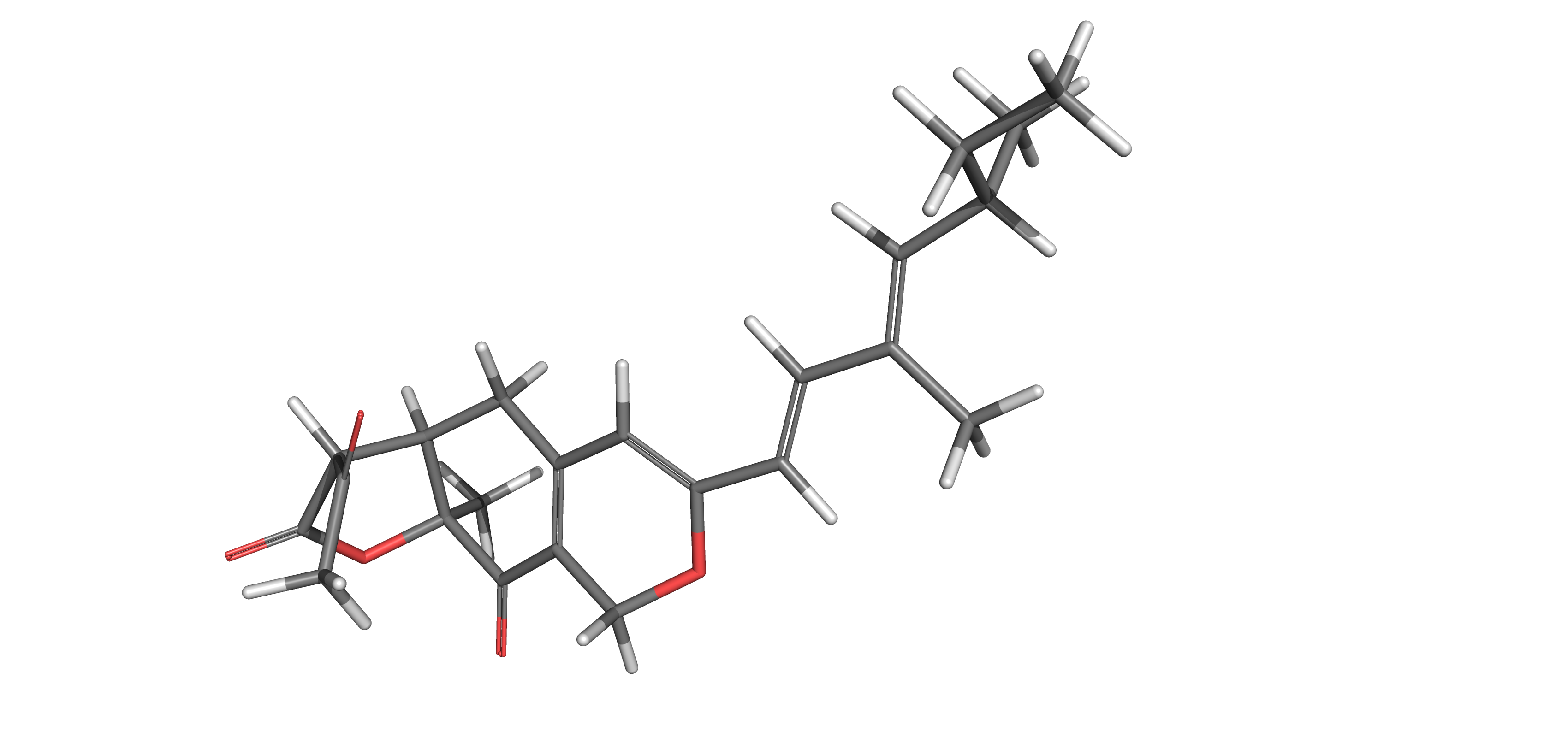

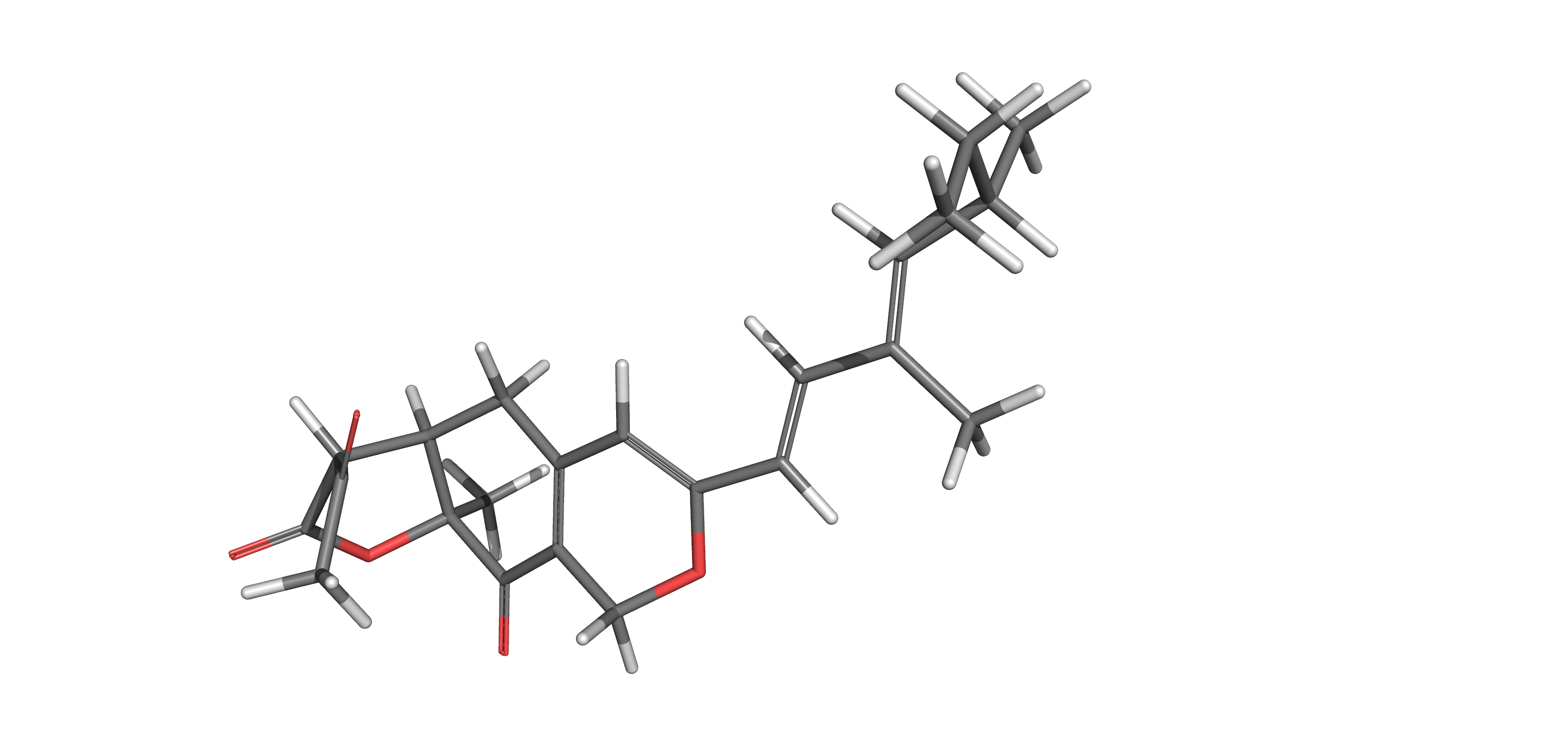


**Conf. 6**

[19.16]

Δ*G* = 2.37

P = 1.15

**Conf. 5**

[15.75]

Δ*G* = 1.64

P = 3.95

Fig. S3-1 The optimized structures of 6 conformers of 1 with the minimum value of frequency [in brackets, cm^−1^], relative free-energy (Δ*G*, kcal/mol), and Boltzmann distribution (P, %), at 298.15 K, calculated at the B3LYP/def2-TZVP level in MeOH.


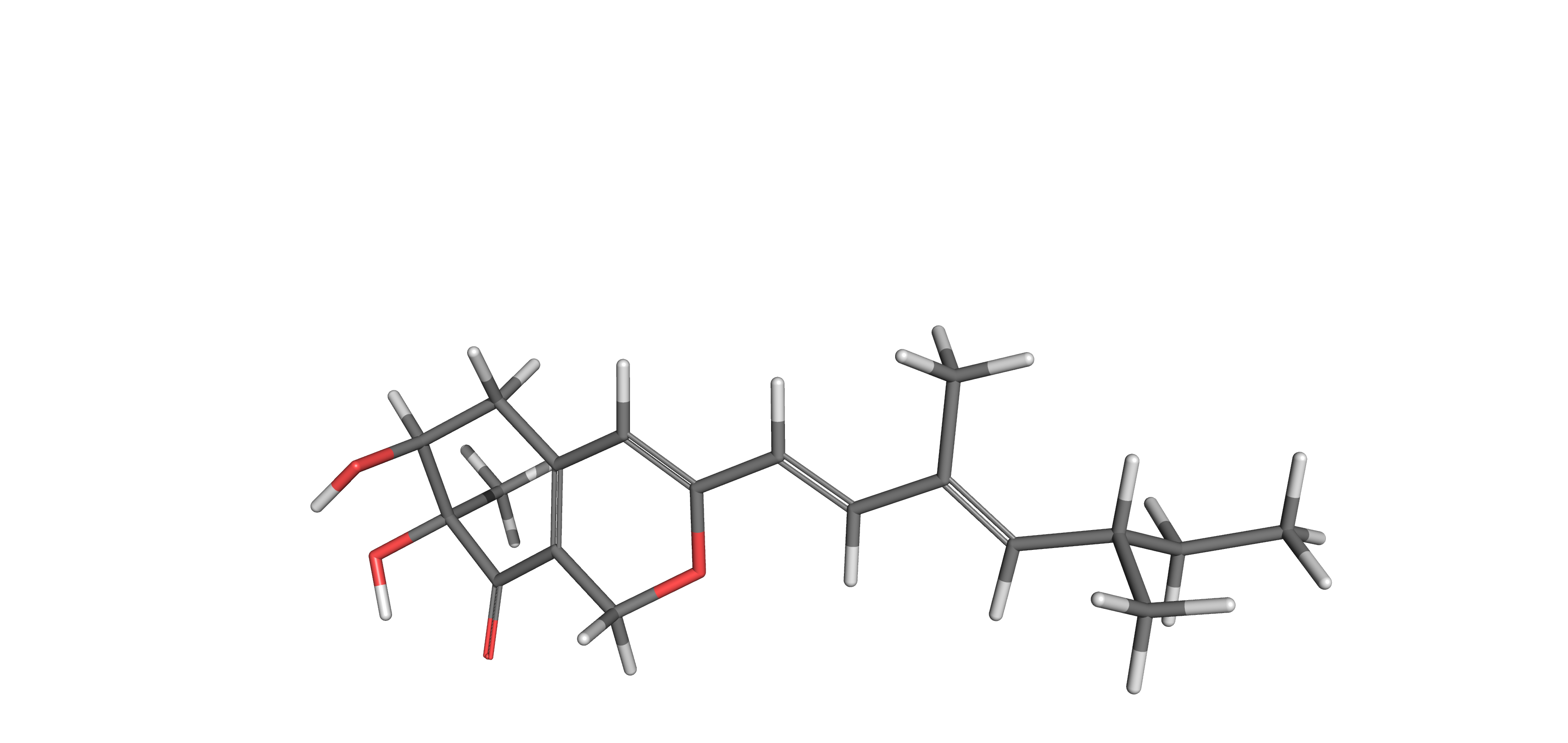

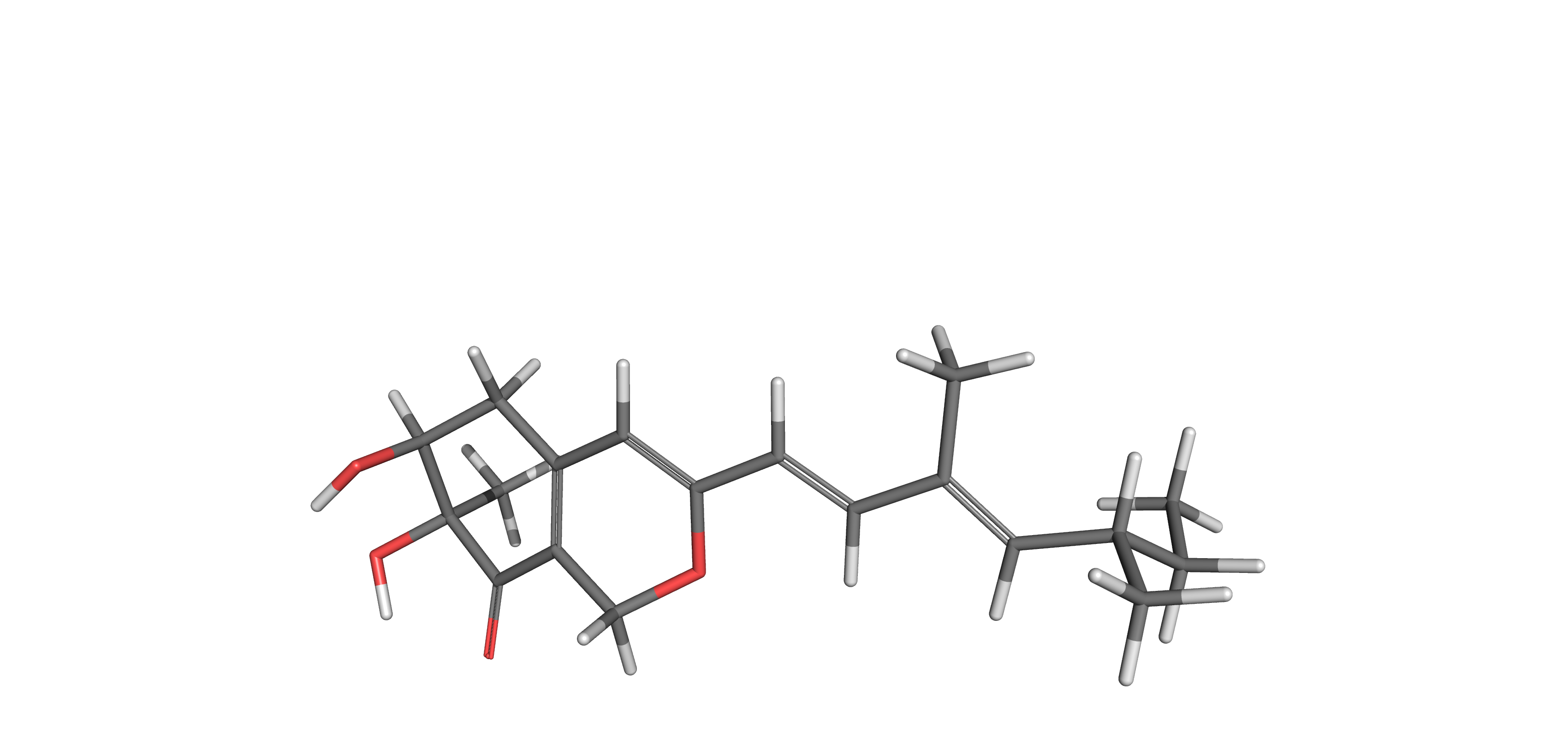


**Conf. 2**

[18.18]

Δ*G* = 0.51

P = 26.70

**Conf. 1**

[15.98]

Δ*G* = 0.00

P = 63.43


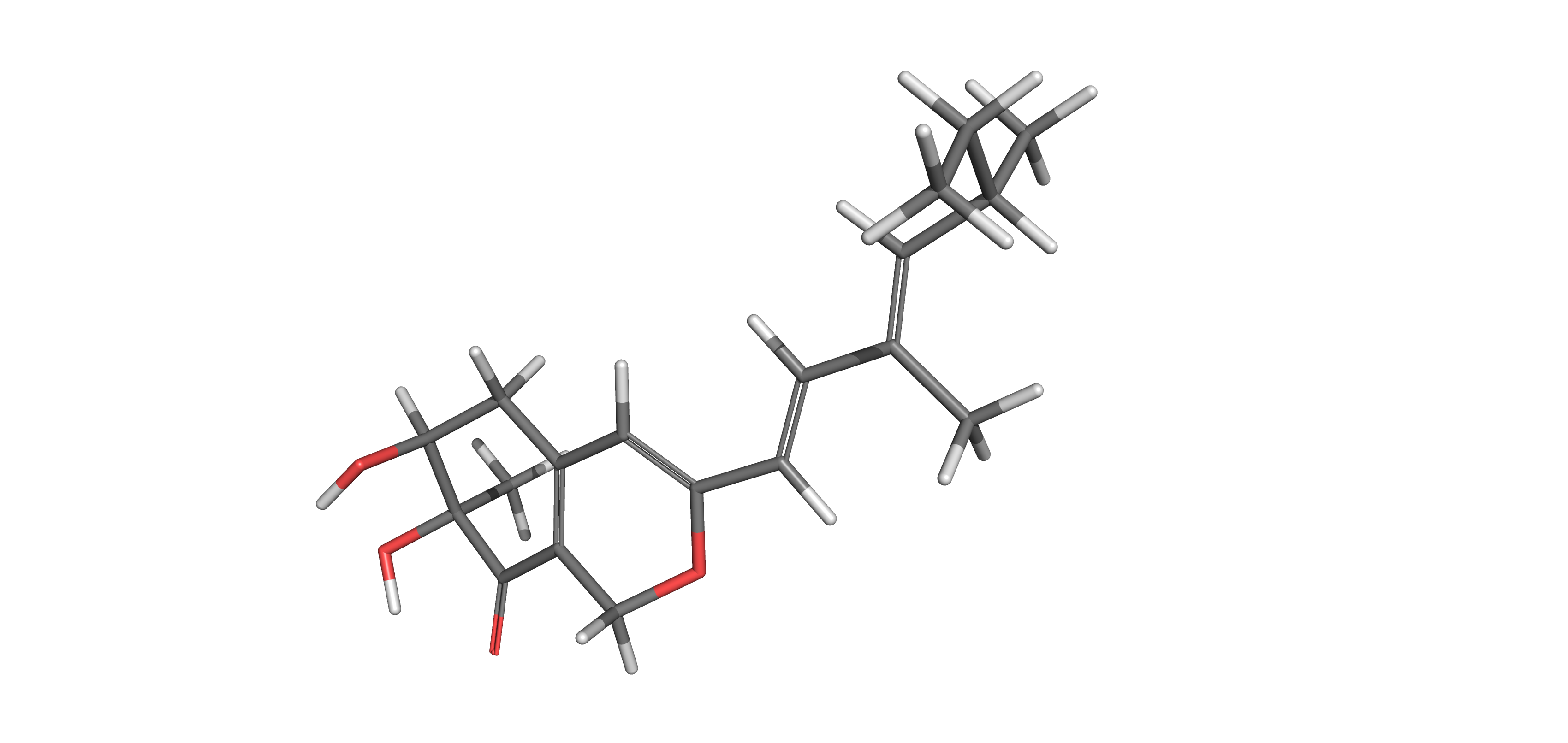


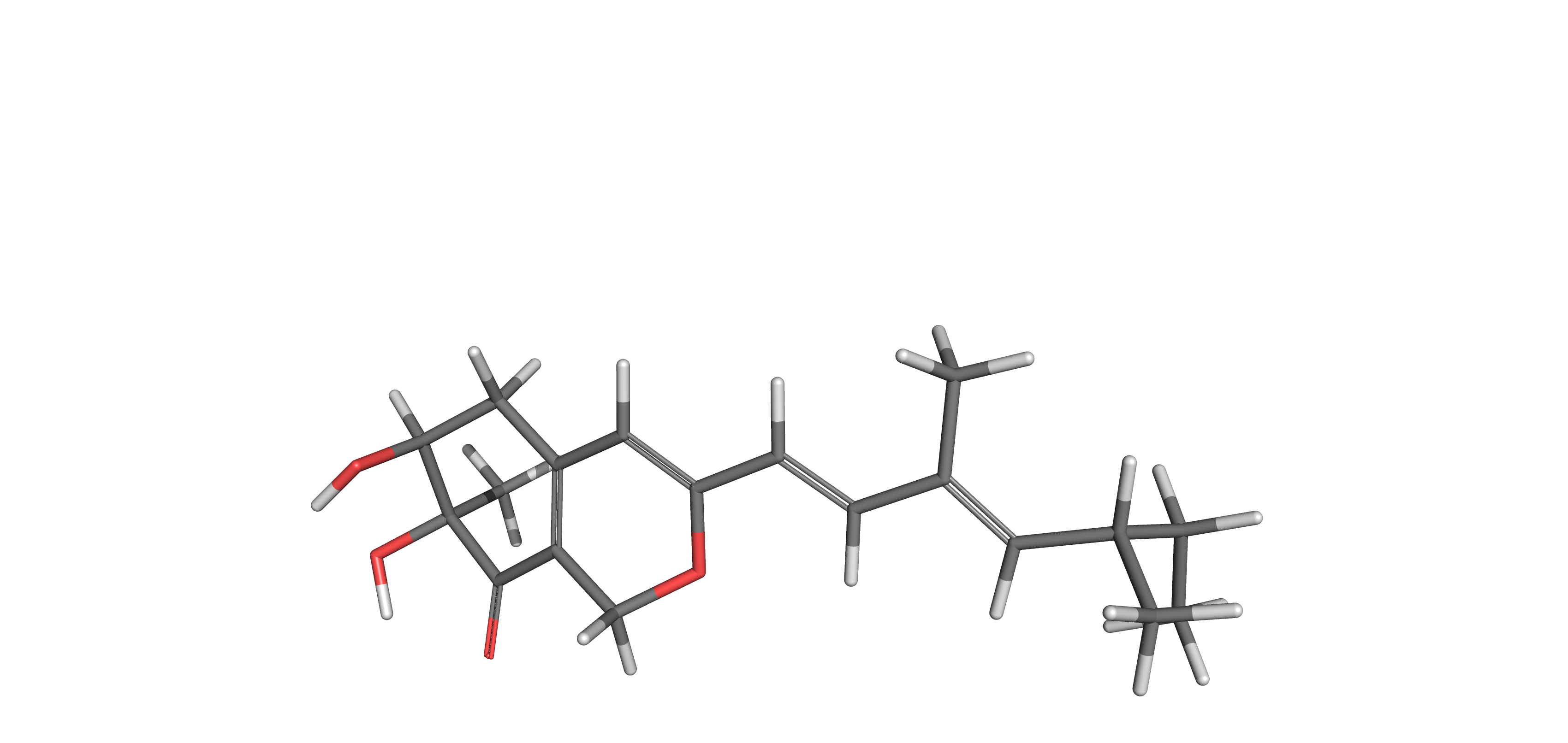


**Conf. 3**

[13.71]

Δ*G* = 1.48

P = 5.19

**Conf. 4**

[15.76]

Δ*G* = 1.74

P = 3.38

Fig. S3-2 The optimized structures of 4 conformers of 2 with the minimum value of frequency [in brackets, cm^−1^], relative free-energy (Δ*G*, kcal/mol), and Boltzmann distribution (P, %), at 298.15 K, calculated at the B3LYP/def2-TZVP level in MeOH.


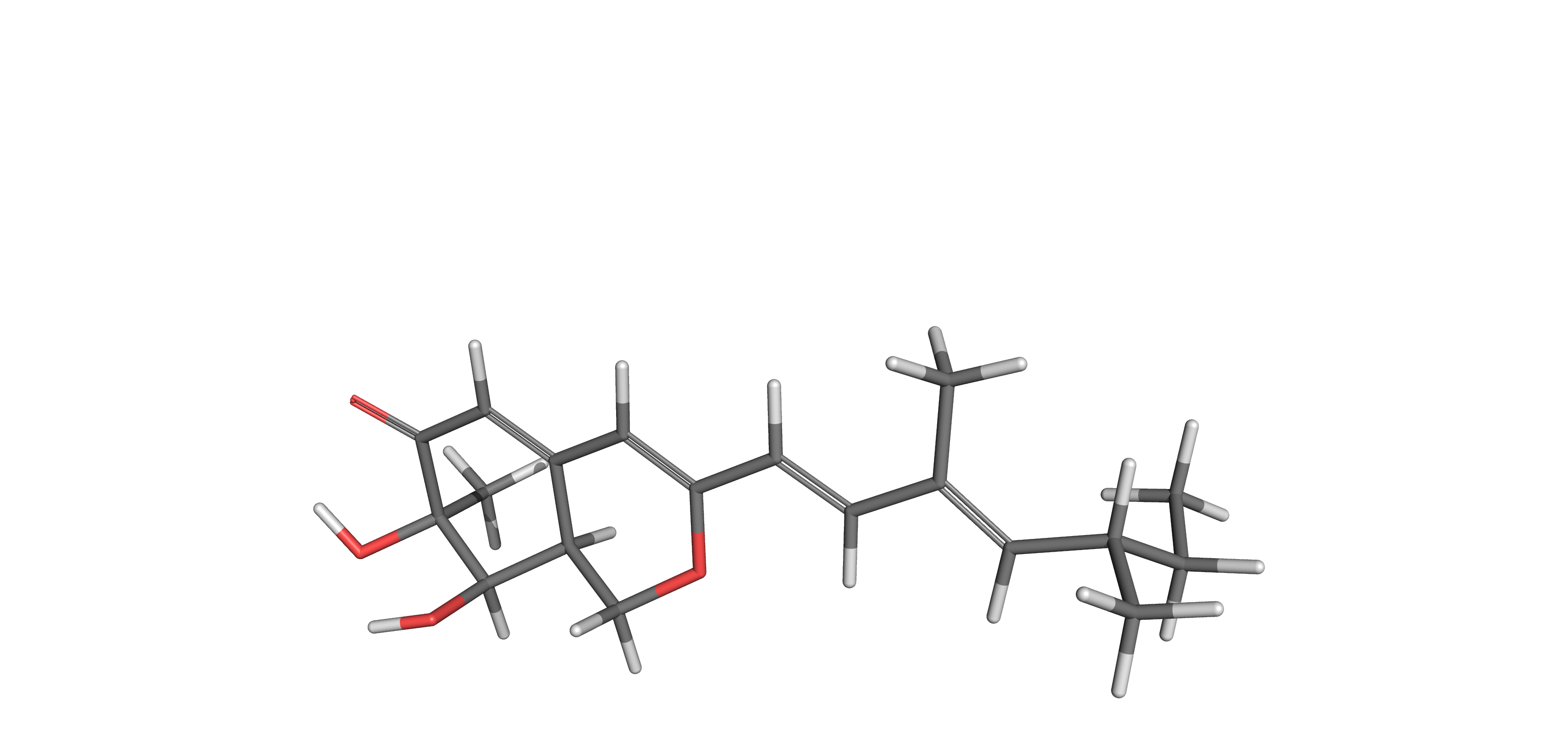

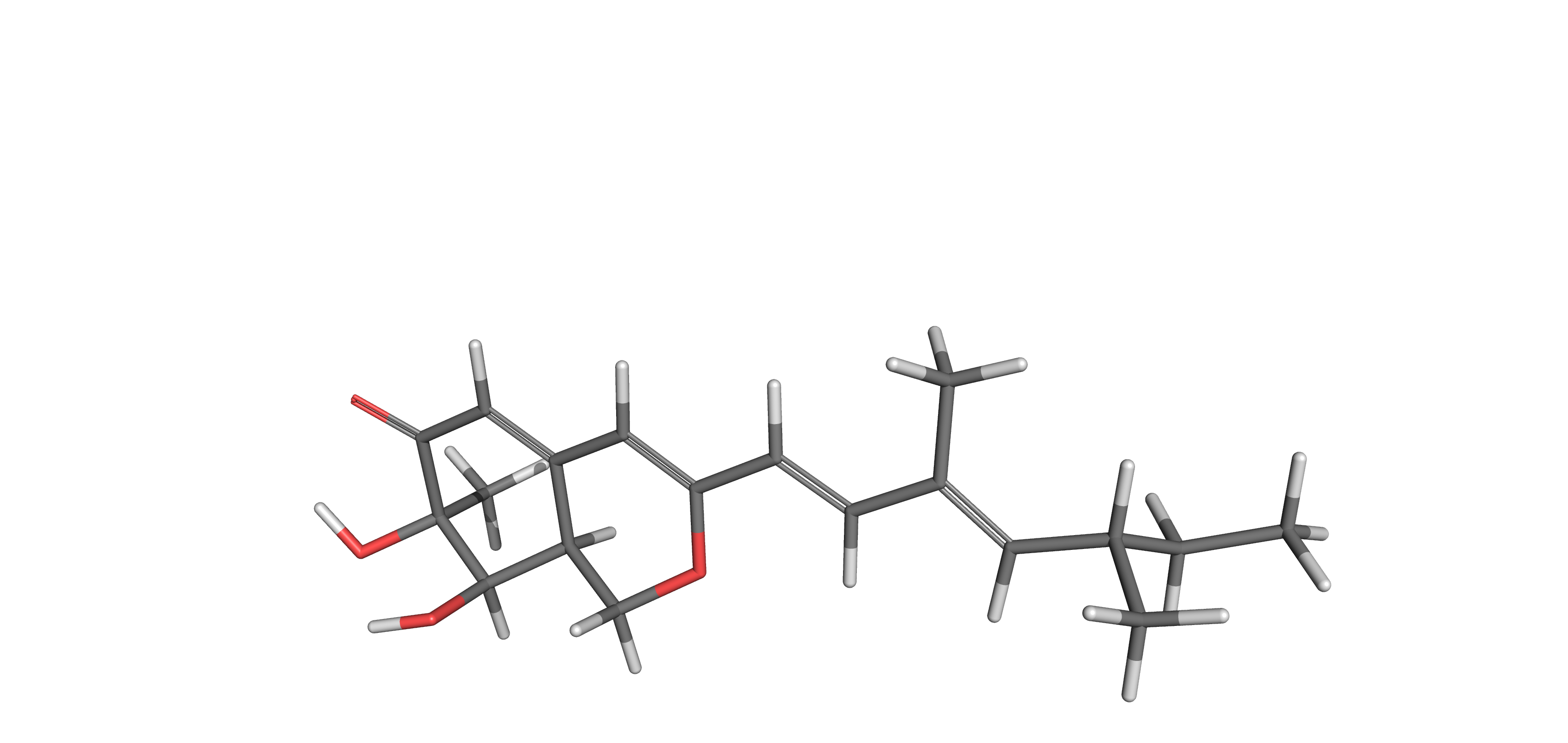


**Conf. 1**

[9.77]

Δ*G* = 0.00

P = 79.37

**Conf. 2**

[16.00]

Δ*G* = 0.94

P = 16.36


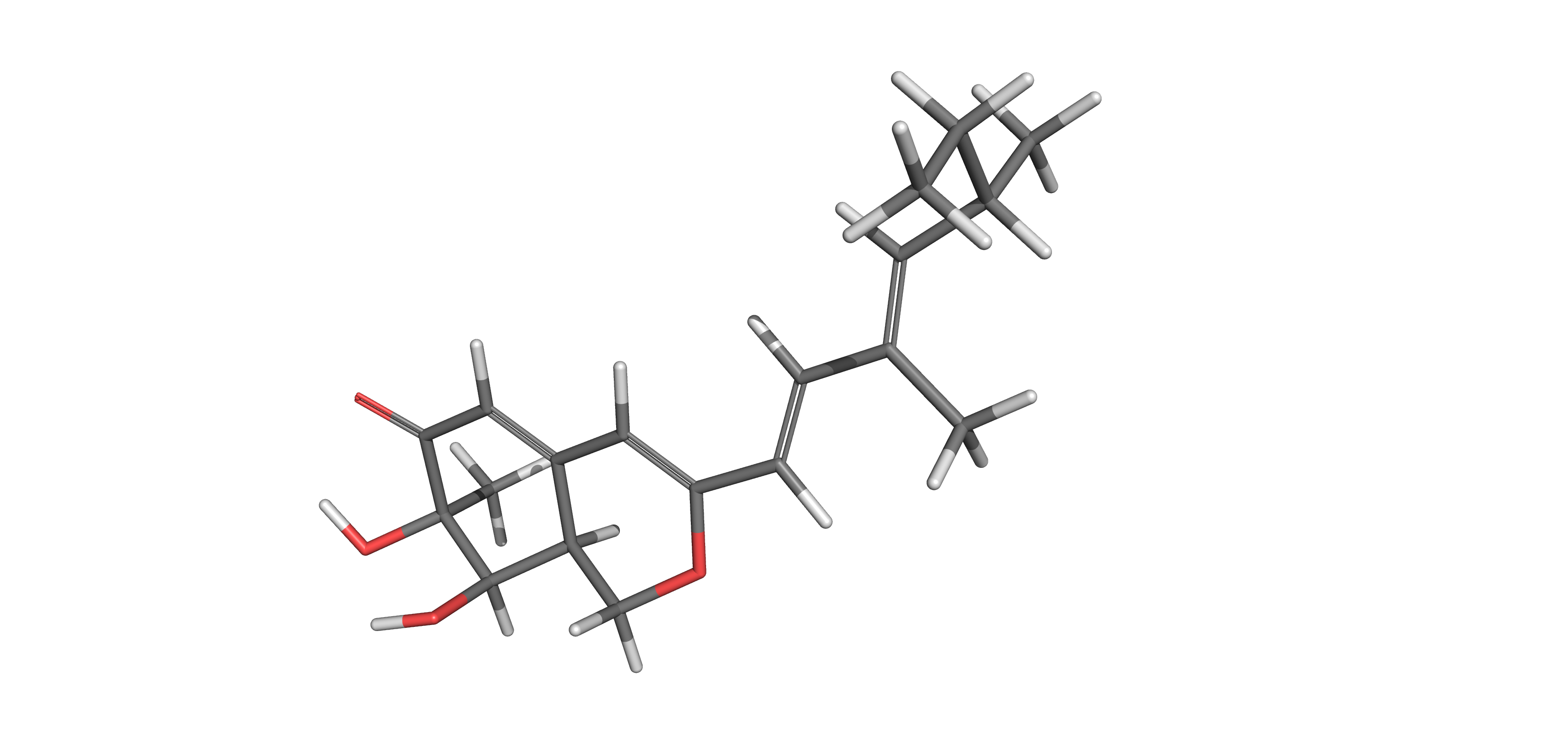


**Conf. 4**

[14.45]

Δ*G* = 2.42

P = 1.34


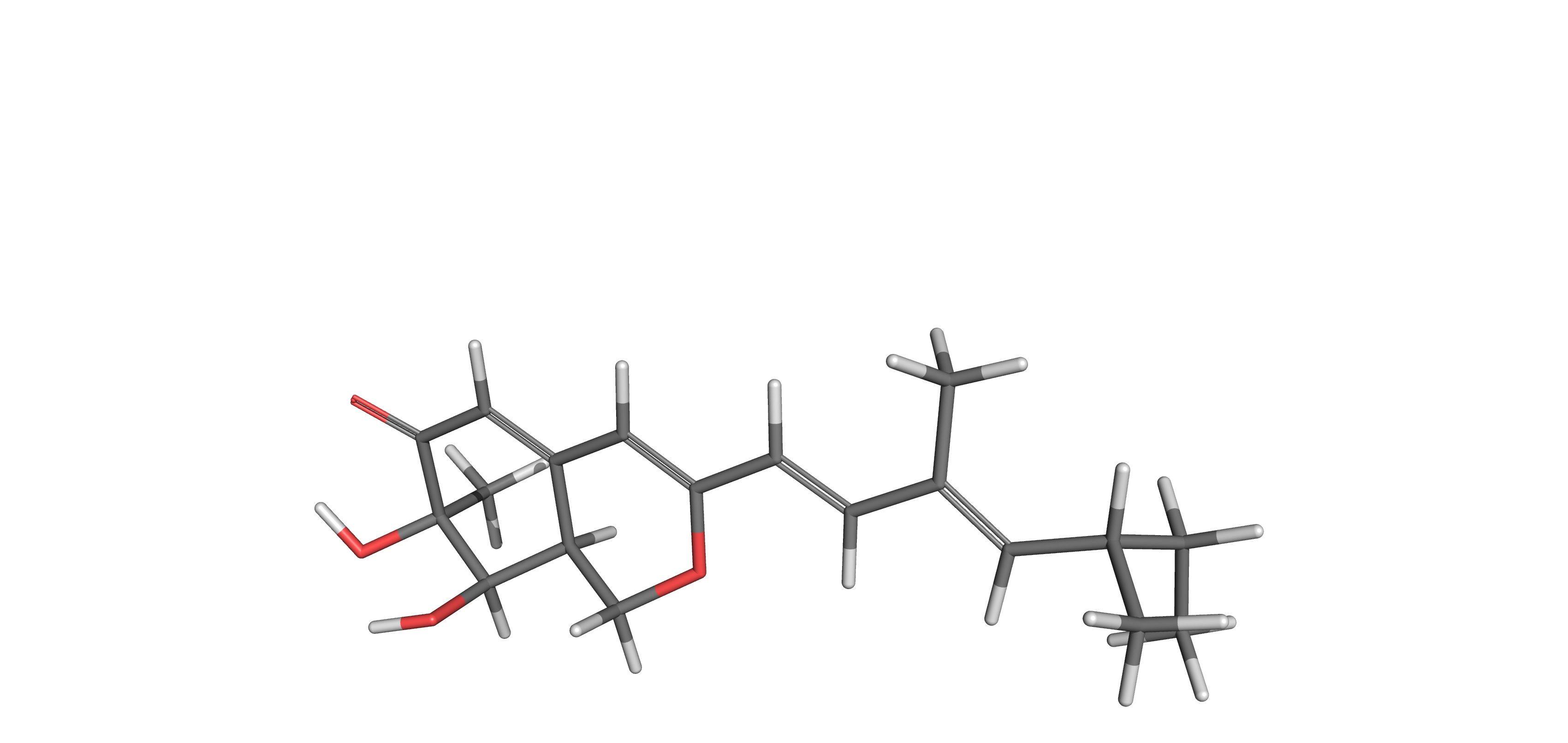


**Conf. 3**

[14.92]

Δ*G* = 2.20

P = 1.93

Fig. S3-3 The optimized structures of 4 conformers of 4 with the minimum value of frequency [in brackets, cm^−1^], relative free-energy (Δ*G*, kcal/mol), and Boltzmann distribution (P, %), at 298.15 K, calculated at the B3LYP/def2-TZVP level in MeOH.

**
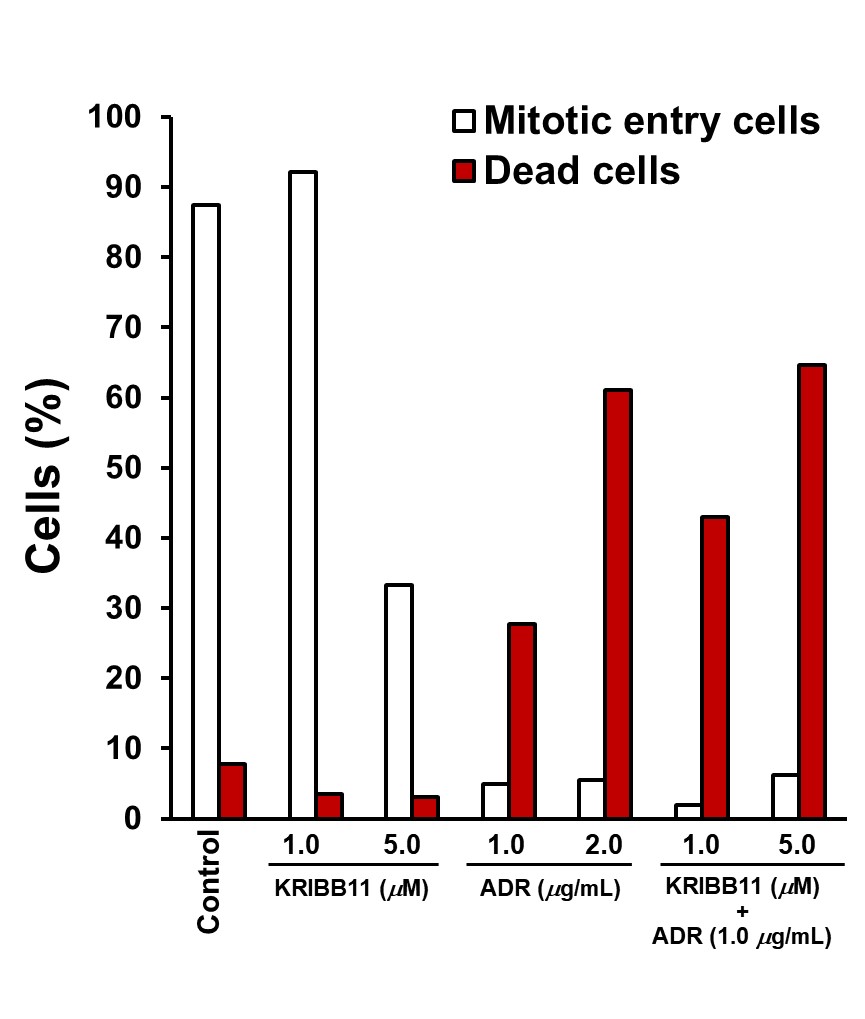
S4. Effects of the KRIBB11 (positive control) on cell proliferation and death.**

Fig. S4 Effects of the KRIBB11 (positive control) on cell proliferation and death. The number of mitotic entry cells and dead cells were counted during time-lapse imaging at one field. Each field captured more than 100 cells. HeLa cells were treated with KRIBB11 (1.0 and 5.0 *μ*M), ADR (1.0 and 2.0 *μ*g/ml), or combination of ADR (1.0 *μ*g/ml) and KRIBB11 (1.0 and 5.0 *μ*M) for 24 h.

**
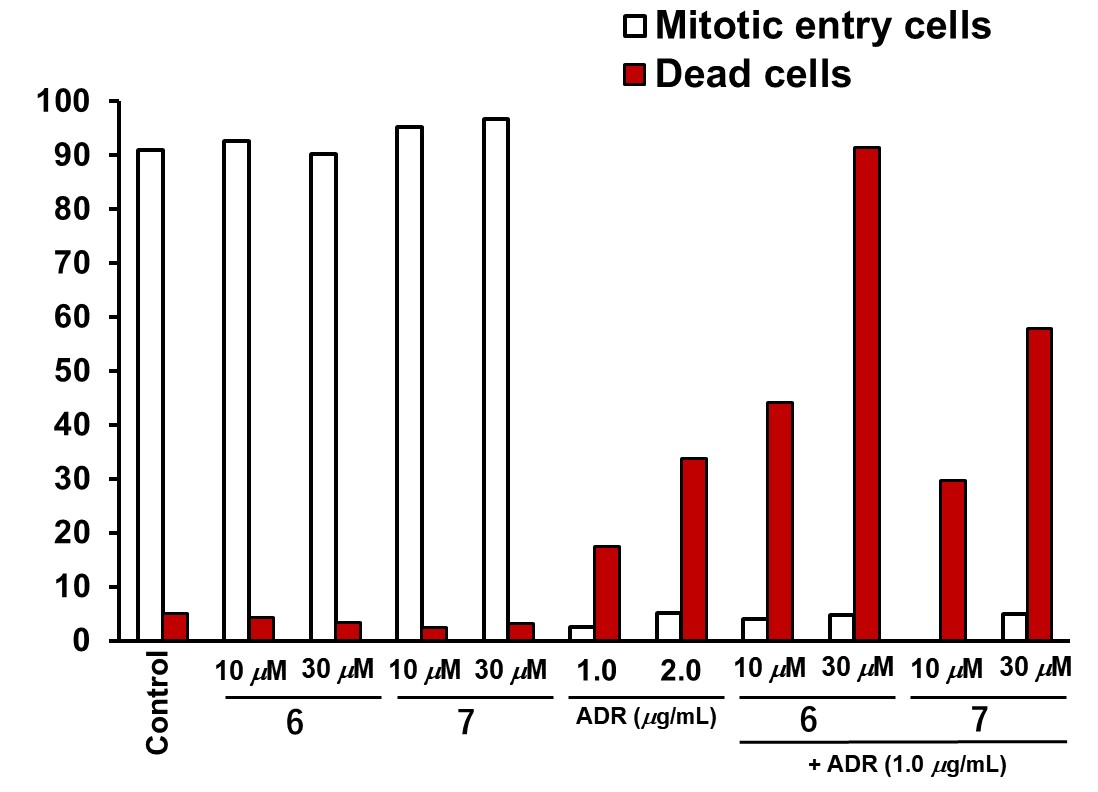
S5. Effects of the compounds 6 and 7 at two concentrations (10 μM and 30 μM) on cell proliferation and death.**

Fig. S5 Effects of the compounds 6 and 7 on cell proliferation and death. The number of mitotic entry cells and dead cells were counted during time-lapse imaging at one field. Each field captured more than 100 cells. HeLa cells were treated with compounds 6 and 7, ADR, or their combinations as indicated concentrations for 24 h.

**S6. The expression of HSP 105, 90, and 70 on HeLa cell treated with 6.**


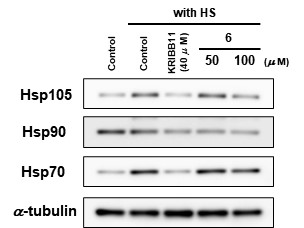


Fig. S6 The expression of Hsp105, 90, and 70 on HeLa cells treated with 6. HeLa cells were treated with 6 or KRIBB11 for 30 min and then treated with heat shock (42 °C) for 1 h and recovered at 37 °C for 5 h.
